# Mycophenolate mofetil versus azathioprine in kidney transplant recipients on steroid-free, low-dose cyclosporine immunosuppression (ATHENA): A pragmatic randomized trial

**DOI:** 10.1371/journal.pmed.1003668

**Published:** 2021-06-24

**Authors:** Piero Ruggenenti, Paolo Cravedi, Eliana Gotti, Annarita Plati, Maddalena Marasà, Silvio Sandrini, Nicola Bossini, Franco Citterio, Enrico Minetti, Domenico Montanaro, Ettore Sabadini, Regina Tardanico, Davide Martinetti, Flavio Gaspari, Alessandro Villa, Annalisa Perna, Francesco Peraro, Giuseppe Remuzzi

**Affiliations:** 1 Istituto di Ricerche Farmacologiche Mario Negri IRCCS, Bergamo, Italy; 2 Unit of Nephrology and Dialysis, ASST Papa Giovanni XXIII, Bergamo, Italy; 3 Division of Nephrology, Icahn School of Medicine at Mount Sinai, New York, New York, United States of America; 4 Unit of Nephrology, ASST degli Spedali Civili di Brescia, Brescia, Italy; 5 Unit of Kidney Transplantation, Fondazione Policlinico Universitario Agostino Gemelli IRCCS, Roma, Italy; 6 Unit of Nephrology, ASST Grande Ospedale Metropolitano Niguarda, Milano, Italy; 7 SOC di Nefrologia, Dialisi e Trapianto Renale della Azienda Ospedaliero Universitaria “S. Maria della Misericordia,” Udine, Italy

## Abstract

**Background:**

We compared protection of mycophenolate mofetil (MMF) and azathioprine (AZA) against acute cellular rejection (ACR) and chronic allograft nephropathy (CAN) in kidney transplant recipients on steroid-free, low-dose cyclosporine (CsA) microemulsion maintenance immunosuppression.

**Methods and findings:**

ATHENA, a pragmatic, prospective, multicenter trial conducted by 6 Italian transplant centers, compared the outcomes of 233 consenting recipients of a first deceased donor kidney transplant induced with low-dose thymoglobulin and basiliximab and randomized to MMF (750 mg twice/day, *n =* 119) or AZA (75 to 125 mg/day, *n* = 114) added-on maintenance low-dose CsA microemulsion and 1-week steroid. In patients without acute clinical or subclinical rejections, CsA dose was progressively halved. Primary endpoint was biopsy-proven CAN. Analysis was by intention to treat.

Participants were included between June 2007 and July 2012 and followed up to August 2016. Between-group donor and recipient characteristics, donor/recipient mismatches, and follow-up CsA blood levels were similar. During a median (interquartile range (IQR)) follow-up of 47.7 (44.2 to 48.9) months, 29 of 87 biopsied patients on MMF (33.3%) versus 31 of 88 on AZA (35.2%) developed CAN (hazard ratio (HR) [95% confidence interval (CI)]: 1.147 (0.691 to 1.904, *p* = 0.595). Twenty and 21 patients on MMF versus 34 and 14 on AZA had clinical [HR (95% CI): 0.58 (0.34 to 1.02); *p* = 0.057) or biopsy-proven subclinical [HR (95% CI): 1.49 (0.76 to 2.92); *p* = 0.249] ACR, respectively. Combined events [HR (95% CI): 0.85 (0.56 to 1.29); *p* = 0.438], patient and graft survival, delayed graft function (DGF), 3-year glomerular filtration rate (GFR) [53.8 (40.6;65.7) versus 49.8 (36.8;62.5) mL/min/1.73 m^2^, *p* = 0.50], and adverse events (AEs) were not significantly different between groups.

Chronicity scores other than CAN predict long-term graft outcome. Study limitations include small sample size and unblinded design.

**Conclusions:**

In this study, we found that in deceased donor kidney transplant recipients on low-dose CsA and no steroids, MMF had no significant benefits over AZA. This finding suggests that AZA, due to its lower costs, could safely replace MMF in combination with minimized immunosuppression.

**Trial registration:**

**ClinicalTrials.gov** NCT00494741; EUDRACT 2006-005604-14.

## Abstract 2

Piero Ruggenenti and co-workers study maintenance immunosuppression in deceased-donor kidney transplantation.

## Author summary

**Why was this study done?:**

- Based on the results of registration trials showing that mycophenolate mofetil (MMF) prevented acute cellular rejection (ACR) more effectively than azathioprine (AZA) in the setting of a regimen including the oil-based cyclosporine (CsA) formulation, MMF progressively replaced AZA as part of standard maintenance immunosuppressive treatment in most kidney transplant centers worldwide.
- However, subsequent academic studies with standard doses of a more stable CsA microemulsion formulation found that MMF offered no benefit over AZA on the incidence of ACR or chronic allograft nephropathy (CAN).
- Whether MMF and AZA are associated with similar long-term graft and patient outcomes also in kidney transplant recipients given antibody induction therapy and maintenance immunosuppression with low-dose CsA without steroids is unknown.

**What did the researchers do and find?:**

- Based on immune studies supporting the concept that combined induction with low-dose thymoglobulin and basiliximab is protolerogenic, in this prospective, randomized trial, we compared the effects of MMF and AZA in 233 recipients of a kidney transplant from a deceased donor who received dual induction and steroid-free maintenance immunosuppression with lower than standard doses of CsA microemulsion.
- We found that during a median follow-up of nearly 4 years, the cumulative incidence of biopsy-proven CAN (primary endpoint) was similar in patients assigned to MMF and in those allocated to AZA therapy.
- Biopsy-proven clinical and subclinical acute rejection, patient and graft survival, delayed graft function (DGF), and renal function recovery were also similar between groups, as was the rate of adverse events (AEs), in particular of those that were treatment related.

**What do these findings mean?:**

- In deceased donor kidney transplant recipients, MMF and AZA share a similar long-term risk/benefit profile, even in the context of minimized maintenance immunosuppressive therapy.
- Considering that MMF is an expensive medication, the preferential use of AZA instead of MMF for maintenance immunosuppression would provide substantial cost saving, without affecting graft outcomes.
- Considering also the reduced costs for low-dose CsA chronic therapy, dual induction combined with steroid-free minimized maintenance immunosuppression, in addition to reduce treatment-related long-term side effects, might facilitate the access to a kidney transplant in particular in resource-limited settings.

## Introduction

Based on results of registration trials showing 30% to 50% reduced 6-month cumulative rates of acute kidney graft rejection with mycophenolate mofetil (MMF) compared to azathioprine (AZA) [1,2] or placebo [3], MMF was launched as part of standard maintenance immunosuppression for organ transplantation [4]. Few years later, however, the MYcofenolate Steroid Sparing (MYSS) trial and the long-term MYSS follow-up study [5,6] found that 5-year cumulative incidence of acute rejection, graft and patient survival, glomerular filtration rate (GFR) decline, new onset of clinical proteinuria—taken as a marker of chronic allograft nephropathy (CAN)—and adverse event (AE) rates were comparable in 334 deceased donor kidney transplant recipients randomized to MMF or AZA in combination with cyclosporine (CsA) and 6-month steroids. A subsequent paired kidney analysis on 476 renal transplant recipients found even more acute rejections with MMF versus AZA, while there was no difference in patient or graft survival between treatment groups [7]. No benefits of MMF over AZA were reported also in recipients of other organ transplants [8]. Notably, 2 large systematic reviews including more than 6,000 kidney transplant recipients altogether [9,10], found that benefits of MMF reported by industry-sponsored studies, were not confirmed in subsequent academic studies. A plausible explanation is that in original registration trials, MMF and AZA were tested in combination with the oil-based CsA formulation [11]. Then, oil-based CsA formulation was progressively replaced by the more rapidly, completely, and reproducibly absorbed CsA microemulsion preparation. CsA microemulsion was approved for use in clinical transplantation in 1994 and soon became the preferred form of CsA. Its introduction in clinical transplantation was associated with a dramatic reduction in the rates of acute rejections without incremental toxicity [11,12]. Considering that MMF was introduced in clinics concurrently with CsA microemulsion, it is conceivable that the perceived benefits of MMF over AZA could be explained by optimized immunosuppression achieved with the novel CsA formulation rather than by a genuine effect of MMF. Consistently, academic trials comparing the antirejection effects of MMF and AZA, in combination with CsA microemulsion, failed to detect any appreciable difference between the 2 medications [5,6,13–17].

Despite the aforementioned findings, analyses of the American Scientific Registry of Transplant Recipients database showed that 95% of patients admitted for kidney transplantation between 1998 and 2006 were on MMF at the time of discharge. The appropriateness of this attitude should be probably reassessed, especially considering that standard doses of MMF (2 g/day) are almost 15 times more expensive than equivalent doses of AZA (100 mg/day). Thus, generalized treatment with MMF (instead of AZA) unnecessarily increases costs for pharmacological prevention of acute rejection by approximately €5,000 (or US$5,400) per patient/year, or by about €2,200 (or US$2,400) per patient/year in the case a generic formulation of MMF is used.

Despite CsA microemulsion background therapy, however, approximately one-half of participants of the MYSS trial had at least 1 acute allograft rejection over 6-year follow-up. Moreover, 6.1% and 6.8% of those randomized to MMF or AZA, respectively, lost their kidneys, and within each treatment groups, 4% of participants died with a functioning graft [5,6]. Antibody induction, steroid-free immunosuppression, and avoidance of calcineurin inhibitors have been suggested to improve these outcomes [18]. In particular antibody induction, in addition to reduce the risk of acute kidney graft rejection [19], even after early steroid withdrawal [20,21], allows CsA tapering and even withdrawal early posttransplant in order to prevent chronic nephrotoxicity without increasing the risk of allograft rejection (reviewed in [22]). To this end, rabbit thymoglobulin (RATG) are the most effective induction therapy but are associated with a high rate of serious AEs (SAEs) [22]. Antibodies against the interleukin 2 (IL-2) receptor are remarkably safer but definitely less effective [23]. Thus, to improve the risk/benefit profile of antibody treatment, we implemented an induction protocol based on low-dose RATG plus anti-IL-2 receptor blocker basiliximab, a regimen that allows to effectively prevent acute rejection while avoiding steroids and using lower than standard doses of CsA microemulsion [24,25]. Based on immune studies supporting the concept that this combined induction is protolerogenic [26], we designed a prospective randomized controlled trial testing the hypothesis that, with dual induction therapy, MMF and AZA are associated with not significantly different long-term graft and patients’ outcomes even in the context of minimized maintenance immunosuppression.

## Methods

### Prospective protocol and analysis plan

ATHENA was “A randomized, prospective, multicentre trial to compare THe Effect on chronic allograft Nephropathy prevention of mycophenolate mofetil versus Azathioprine as the sole immunosuppressive therapy for kidney transplant recipients.” This academic Phase III parallel clinical trial was primarily aimed at comparing the cumulative incidence of CAN in recipients of a first kidney transplant from a deceased donor who were randomized to receive 750 mg of MMF twice daily (*n =* 119) or 75 mg (125 mg if body weight >75 kg) once daily of AZA (*n* = 114) in combination with low-dose RATG and basiliximab dual induction and maintenance immunosuppression with CsA microemulsion and no steroids. Treatment allocation was open, but all study assessors were blinded to treatment. In consenting recipients without biopsy-proven acute clinical rejection or subclinical rejection at 12 to 18 months per-protocol surveillance graft biopsy, CsA microemulsion dose was progressively tapered. According to initial study design, CsA had to be progressively tapered up to withdrawal. However, because this approach was found to associate with excess risk of acute rejections, in September 2011, the study was amended specifying that CsA dose could not be reduced to less than half of the previous maintenance dose.

### Participants, setting, and ethics statement

Participants were identified among renal transplant recipients referred to the nephrology units and/or transplant centers of 6 hospitals in Italy coordinated by the Department of Renal Medicine of the Clinical Research Center (CRC) for Rare Diseases “Aldo e Cele Daccò” Villa Camozzi of the Istituto di Ricerche Farmacologiche Mario Negri IRCCS (Bergamo, Italy; see ATHENA study organization in S1 Text).

Adult (≥18 years) male and female recipients of a first single or double kidney transplant from a deceased donor were eligible. We excluded patients with specific contraindications to RATG therapy such as severe leukopenia (white blood cell (WBC) <2,000/mm^3^), high immunological risk because of a second transplant or panel reactivity ≥10%, history of malignancy (except nonmetastatic basal or squamous cell carcinoma of the skin that had been treated successfully), serological evidence of active hepatitis C or hepatitis B virus infection or human-acquired immunodeficiency virus infection, and any chronic clinical conditions that could affect completion of the trial or confound data interpretation; pregnant, childbearing, or potentially childbearing women without adequate contraception; and patients who did not fully understand the purposes of the study and relative follow-up or were already involved in other studies (for further details, please see Study protocol in S2 Text and https://clinicaltrials.gov/ct2/show/NCT00494741).

The study protocol was approved by each site’s institutional review board according to the Good Clinical Practice guidelines. Written informed consent was obtained from all participants and study conduct was in compliance with the 2004 Declaration of Helsinki (revised on 2008 and 2013). Data were locally recorded in dedicated electronic case report forms and centralized into the database at the coordinating center. Data were periodically reviewed by a safety review board including 2 nephrologists and 1 statistician who were not involved in study conduct (see S1 Text). This study is reported as per the Consolidated Standards of Reporting Trial (CONSORT) guideline (see CONSORT checklist in S3 Text).

### Randomization

Eligible participants were randomly assigned 1:1 to receive oral treatment with 750 mg of MMF (CellCept, Roche) twice daily or with 75 mg of the galenic formulation of AZA (125 mg if body weight >75 kg) once daily by an independent investigator (G.A.G., see: ATHENA study organization in S1 Text), using a web-based, computer-generated randomization list created using SAS (version 9.2), stratified by center with random block size of 4 and 8. Randomization was centralized at the Laboratory of Biostatistics of the CRC.

### Baseline assessment and procedures

Biopsy samples were obtained from kidney grafts before reperfusion for evaluation of tubular, interstitial, vascular, and glomerular changes. After baseline evaluation of recipients’ demographic and clinical characteristics and recording of causes of graft failure, duration of chronic dialysis therapy, donors’ main characteristics and donor/recipient HLA A, B, and DR mismatches, all study participants received induction therapy with RATG (0.5 mg/kg from the day of transplant up to day 6) and basiliximab (20 mg before surgery and at day 4 after transplantation). One pulse of 500 mg of methylprednisolone was infused the day of transplant (day 0), and 2 additional pulses of 250 mg and 125 mg were infused at posttransplant days 1 and 2, respectively. Thereafter, patients were steroid free. CsA (Neoral, Novartis, Basel, Switzerland) daily doses were titrated to achieve and maintain trough CsA levels between 300 and 400 ng/mL during the first week after transplantation, 200 and 250 ng/mL up to month 2, 150 and 200 ng/mL from month 3 to month 4, and 100 and 150 ng/mL thereafter. The day of transplant participants were randomly assigned to the MMF or AZA treatment arm. Maintenance doses were reduced in case of WBC count lower than 2,000/mm^3^ and whenever deemed clinically appropriate. All patients were treated and monitored according to protocol guidelines.

Between months 12 to 18 after transplant, a surveillance biopsy was obtained from consenting patients without previous clinical acute rejections or excess bleeding risk or other contraindications in order to evaluate the presence of histological changes consistent with subclinical rejection, disease recurrence, or de novo glomerulonephritis. These events were treated as per center practice and no change was introduced in CsA dose of affected patients. Conversely, in consenting patients without events, CsA dose was reduced by about 10% of the initial dose every 4 weeks in order to achieve 50% CsA dose tapering during 20 to 24 weeks after transplant (S2 Text). Deviations from this schedule were allowed as deemed appropriate by the investigators. After completion of at least 3 years of follow-up after randomization, a second surveillance biopsy was planned in order to evaluate the cumulative incidence of biopsy-proven CAN in consenting patients without already established histological diagnosis of CAN. The final decision to perform the surveillance biopsy was left to the clinical judgment of the physicians in charge of the study participants. If they concluded that the biopsy was not indicated because the possibility to detect changes consistent with CAN was negligible because kidney function was normal and stable and there was no proteinuria, they were allowed not performing the biopsy provided their decision was discussed with the first investigator and justified in patient CRF.

All biopsies were evaluated at each center to guide patient management according to clinical judgment and protocol guidelines and then centrally reviewed in accordance with the chronic allograft damage index (CADI) and Revised Banff 1997 Classification [27] by 2 pathologists (ES and RT) who had specific expertise in kidney transplant pathology and were blinded to patient treatment. CAN was defined as a CADI score of 2 or more [28]. C4d staining was performed by immunohistochemistry on paraffin sections and scored as 0 (negative), 1 (minimal), 2 (focal), or 3 (diffuse). The pathologists also reported the presence of lesions suggestive of antibody mediated rejection: (1) peritubular capillaritis (ptc score >0); (2) glomerulitis (g score >0); (3) capillary microthrombi; (4) transplant glomerulopathy (cg score >0); and (5) severe intimal arteritis (v score of 3). In biopsies showing signs of recurrent or de novo glomerulonephritides, g and cg scores were not documented.

### Outcome measures

Primary outcome variable was the cumulative incidence of biopsy-proven CAN during the whole observation period. Secondary outcome variables were the cumulative incidence of biopsy-proven acute clinical rejections, the combined outcome of biopsy-proven clinical and subclinical rejections, patient and graft survival, and AEs during the whole observation period. Exploratory outcome variables were histological changes including evidence of subclinical rejection at surveillance biopsies performed 12 to 18 months after transplantation and time-dependent changes in GFR estimated by MDRD formula (eGFR).

For clarity, we decided to report results of comparative analyses between the 2 treatment groups, whereas results of comparative analyses between participants who tapered or did not taper CsA dose will be described separately.

### Sample size and statistical analyses

At the time the study protocol was designed, there were no data from randomized clinical trials on 3-year incidence of biopsy-proven CAN. However, in a 1-year trial of 71 renal transplant recipients randomized to MMF or AZA in combination with steroid and CsA [15], biopsy-proven CAN was reported in 17 of the 37 patients on MMF (46%) and 24 of the 34 on AZA (71%). Assuming, conservatively, a similar incidence of events at 3 years, we calculated that kidney graft biopsies had to be obtained from at least 70 patients per treatment group in order to give the trial an 80% power to detect by a two-sided test (alpha = 0.047, log-rank test, two-sided test), the expected between-group difference in biopsy-proven CAN incidence during the observation period. Assuming that biopsy data could not be obtained from at least 30% of study patients because of contraindications, consent deny to the procedure, or other reasons, we calculated that at least 100 patients per group had to be included. To account for an expected 10% rate of dropouts, we planned to include 112 patients per group for a total of 224 randomized patients. There were 2 interim analyses, on July 2012 and on September 2014. The Method of O’Brien and Fleming was used to determine the threshold for statistical significance at the interim evaluation. For the first and second analyses, the threshold was a *P* value of 0.0006 and of 0.015 (nominal significance level), respectively. For the final analysis, a *P* value of 0.047 or less was used, in order to “preserve” an overall 5% level of significance.

The entire study was analyzed by intention-to-treat principle for endpoint analyses and with a modified intention-to-treat approach for continuous variables in all participants who had at least 1 efficacy measurement after randomization, without imputation of missing data.

Cumulative event curves were described using the Kaplan–Meier procedure, and the Cox regression model was used to estimate the hazard ratio (HR) with the corresponding 95% confidence interval (CI) for the primary and secondary efficacy variables (i.e., CAN and acute rejection, respectively). Participants who did not reach the outcome of interest were considered as right-censored. Proportionality assumptions were assessed using Schoenfeld residuals. Changes in continuous variables were assessed by analysis of covariance (ANCOVA), adjusted for baseline measurement only. All remaining secondary and exploratory efficacy and safety evaluations were carried out using unpaired *t* test, Wilcoxon rank-sum test, chi-squared test, or Fisher exact test (for between-group comparisons) as appropriate. No imputation method was used for missing values. Results were expressed as mean ± standard deviation (SD), median (interquartile range (IQR)), or number (percent). We used SAS version 9.4 and Stata version 15 for all the analyses.

## Results

### Patients

**Fig 1:**
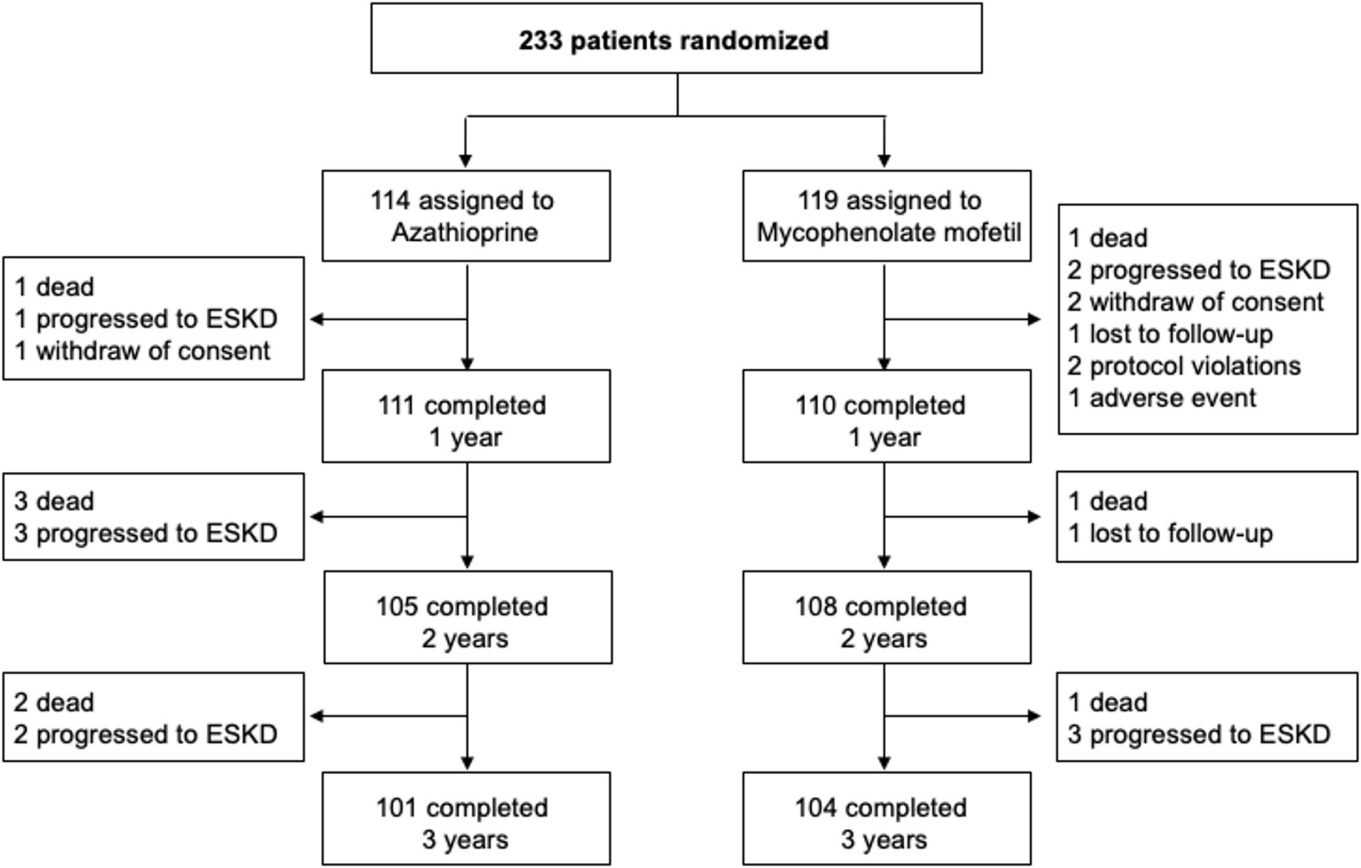
Participant flowchart. ESKD, end-stage kidney disease.

Between June 2007 and July 2012, the participating centers of Bergamo, Brescia, Roma, Milano, Padova, and Udine (see S1 Text) included and randomized 130, 52, 24, 16, 7, and 4 consenting patients, respectively. Of the 233 study participants, 119 were randomized to MMF and 114 to AZA (Fig 1). After randomization, patients were followed up to August 2016 for a median (IQR) of 47.7 (44.2 to 48.9) months. At 13.9 (11.1 to 15.9) months after transplant, a per-protocol surveillance biopsy was obtained from 110 (55 per treatment group) of randomized participants (47.2%) to assess the presence of subclinical rejection, recurrent kidney disease, or de novo glomerulopathy on the graft. Surveillance biopsy at 1 year was not performed in 43 patients (23 on MMF and 20 on AZA) because of previously established diagnosis of acute rejection. Nine participants denied their informed consent to the procedure. In the remaining 71 participants, the biopsy was not performed because of concomitant antiplatelet or anticoagulant therapy (*n =* 27), clinical reasons (*n* = 13), or other reasons (*n* = 31). After surveillance biopsy, CsA dose was tapered in 50 patients (21.5%): 25 per treatment group. In the remaining 60 patients, CsA dose was not modified because of changes detected at surveillance biopsy or patient consent deny. Thirty-seven patients on AZA (32.5%) were switched to MMF, and 11 MMF patients (9.2%) were switched to AZA in most cases because of specific events that in investigator’s judgments were related to patient’s original treatment.

During the follow-up period, 9 patients (3 on MMF and 6 on AZA) died with a functioning graft, and 11 (5 on MMF and 6 on AZA) resumed dialysis therapy because of graft failure. Three additional patients withdrew their consent to continue the study, 2 were lost to follow-up, 2 were withdrawn by the investigators because of major protocol violations, and 1 because of an AE. Thus, 205 of 233 randomized patients (88.0%) were an active follow-up for at least 3 years (Fig 1).

### Baseline characteristics

Donor and recipient characteristics at study inclusion were similar between treatment groups (Table 1). In particular, the distribution of causes of end-stage kidney disease (ESKD) and the proportion of recipients of a single or dual transplant were similar between groups as well as the number of HLA A, B, or DR mismatches between recipients and their corresponding donors (Table 1). At the time of transplant, all patients had a negative T and B cell cross-match with their corresponding donor. Histological changes observed at preimplantation biopsy samples obtained from kidneys allocated to 92 recipients (47 in the MMF and 45 in the AZA group) were similar between the 2 treatment arms (Table 2).

**Table 1:** Baseline characteristics in the study group as a whole (Overall) and according to randomization arm.

	Overall *(n = 233)*	AZA *(n = 114)*	MMF *(n = 119)*
**Recipients**			
Female, *n (%)*	71 (30)	30 (26)	41 (34)
Age, *years*	52.4 ± 12.5	52.5 ± 12.5	52.4 ± 12.5
Weight, *kg*	69.9 ± 13.7	71.6 ± 13.2	68.1 ± 14.0
BMI, *kg/m*^*2*^	24.2 ± 3.9	24.4 ± 3.8	23.9 ± 4.1
Double kidney graft, *n (%)*	20 (8.6)	9 (7.9)	11 (9.2)
Systolic blood pressure, *mm Hg*	138.1 ± 24.7	137.2 ± 21.6	139.0 ± 27.5
Diastolic blood pressure, *mm Hg*	81.7 ± 12.5	81.6 ± 11.6	81.9 ± 13.3
Hypertension, *n (%)*	76 (41.5)	41 (45.6)	35 (37.6)
PRA >20%, *n (%)*	2 (0.9)	2 (1.8)	0 (0.0)
Duration of dialysis, *months*	53.3 ± 34.2	51.6 ± 28.4	55.0 ± 39.0
**Primary cause of renal failure, *n (%)***			
Diabetes mellitus	4 (1.7)	2 (1.8)	2 (1.7)
Glomerulonephritis	52 (22.3)	24 (21.1)	28 (23.5)
Hypertension, renovascular disease	18 (7.7)	10 (8.8)	8 (6.7)
Polycystic kidney disease	50 (21.5)	27 (23.7)	23 (19.3)
Pyelonephritis/Interstitial nephritis	5 (2.1)	4 (3.5)	1 (0.8)
Systemic disease	6 (2.6)	2 (1.8)	4 (3.4)
Urinary tract alteration	15 (6.4)	5 (4.4)	10 (8.4)
Other	36 (15.5)	13 (11.4)	23 (19.3)
Uncertain	47 (20.2)	27 (23.7)	20 (11.6)
**Donors**			
Female, *n* (*%*)	113 (48.5)	57 (50.0)	56 (47.1)
Age, *years*	51.2 ± 14.9	50.1 ± 14.9	52.3 ± 14.4
Weight, *kg*	72.1 ± 16.8	71.3 ± 17.7	72.9 ± 15.8
HLA A mismatches, *n (%)*			
0	38 (16.3)	18 (15.8)	20 (16.8)
1	124 (53.2)	64 (56.1)	60 (50.4)
2	71 (30.5)	32 (28.0)	39 (32.8)
HLA B mismatches, *n (%)*			
0	30 (12.9)	16 (14.0)	14 (11.8)
1	100 (42.9)	46 (40.4)	54 (45.4)
2	103 (44.2)	52 (45.6)	51 (42.9)
HLA DR mismatches, *n (%)*			
0	31 (13.3)	14 (12.3)	17 (14.3)
1	122 (52.4)	62 (54.4)	60 (50.4)
2	80 (34.3)	38 (33.3)	42 (35.3)

Data are number (percent) or mean ± SD.

AZA, azathioprine; BMI, body mass index; HLA, human leukocyte antigen; MMF, mycophenolate mofetil; PRA, panel reactive antibody; SD, standard deviation.

**Table 2:** Histological score at baseline graft surveillance biopsies in the study group considered as a whole (Overall) and in the 2 treatment groups.

	Overall *(n = 92)*	AZA *(n = 45)*	MMF *(n = 47)*
Interstitial fibrosis	0.76 ± 0.59	0.79 ± 0.56	0.73 ± 0.61
Tubular atrophy	0.59 ± 0.61	0.57 ± 0.63	0.60 ± 0.60
Glomerular sclerosis	0.70 ± 0.55	0.58 ± 0.56	0.81 ± 0.52
Arterial narrowing	0.52 ± 0.59	0.50 ± 0.55	0.55 ± 0.63
Tubular necrosis	0.02 ± 0.16	0.00 ± 0.00	0.05 ± 0.22
Score	1.34 ± 1.08	1.36 ± 1.06	1.33 ± 1.12

Score: interstitial fibrosis + tubular atrophy. Data are mean ± SD.

AZA, azathioprine; MMF, mycophenolate mofetil; SD, standard deviation.

### Primary outcome

**Fig 2:**
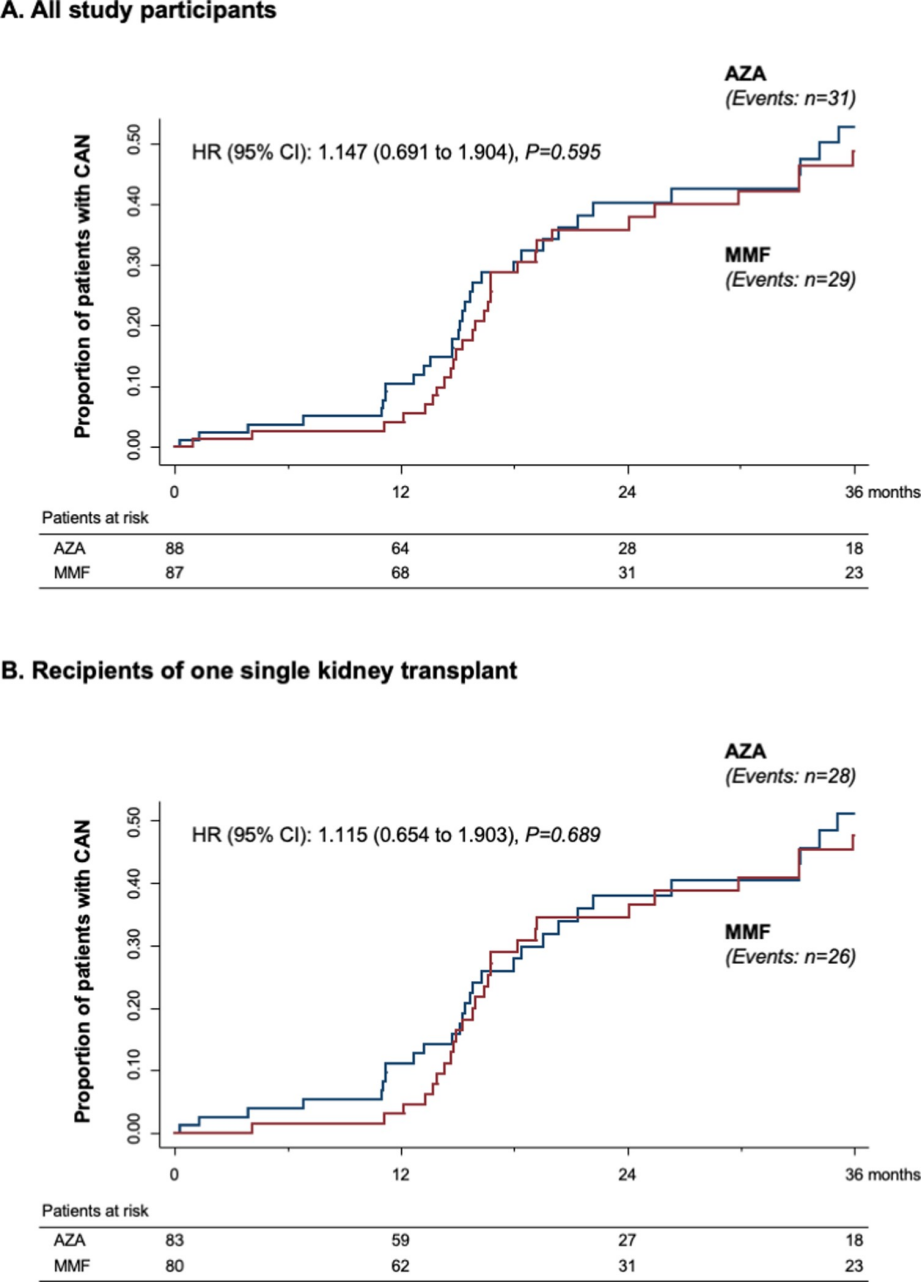
Kaplan–Meier curves of the percentages of patients with CAN in the 2 randomization arms. Kaplan–Meier curves of the percentages of patients with CAN during the 3 years of follow-up in the MMF (red line) and in the AZA (blue line) groups in the overall patient population (**A**) and in patients who received a single kidney transplant (**B**) considered separately. AZA, azathioprine; CAN, chronic allograft nephropathy; CI, confidence interval; HR, hazard ratio; MMF, mycophenolate mofetil.

Histological changes consistent with the diagnosis of CAN were observed in 60 of 175 patients (34.3%) who had at least 1 graft biopsy performed during the study period. CAN was observed in 29 of 87 biopsied patients on MMF (33.3%) and in 31 of 88 biopsied patients on AZA (35.2%). Thus, the cumulative incidence of CAN in patients with histological evaluation was not significantly different [HR (95% CI): 1.147 (0.691 to 1.904), *p* = 0.595] between treatment groups (Fig 2A). CAN was detected in 54 of 163 biopsied patients who received a single transplant (33.1%): 26 patients were on MMF (32.5%) and 28 on AZA (33.7%). Thus, the cumulative incidence of CAN did not differ between treatment groups [HR (95% CI): 1.115 (0.654 to 1.903), *p* = 0.689], even in the subgroup of recipients of single transplants considered separately (Fig 2B). Among the 20 recipients of dual transplants, histological evaluation was available for 7 patients on MMF and 5 patients on AZA. Within each treatment group, there were 3 patients with histological evidence of CAN. A surveillance kidney biopsy at 3 years could not be obtained from 58 patients: 32 on MMF and 26 on AZA. Five of these patients had died, 2 had progressed to ESKD, 6 had been transferred to other centers, 18 were on antiplatelet or anticoagulant therapy, 1 had renal graft cysts, and 6 denied their consent to the procedure. In 14 of the remaining 20 patients, the GFR was progressively improving from month 6 to month 36, and urinary protein excretion was persistently <0.5 g/24 hours. In these cases, the investigators considered that the procedure was not indicated because of the extremely low probability to detect histological changes consistent with CAN. In the remaining 6 cases, there was a protocol violation.

### Secondary outcomes

#### Histological lesions at first surveillance biopsy

Surveillance biopsy samples for evaluations of graft histological changes at 12 to 18 months after transplantation were available from 110 of 233 study patients (47.2%): 55 of 119 patients (46.2%) were on MMF and 55 of 114 (48.2%) on AZA. Glomerular, vascular, tubular, and interstitial changes as well as global histological scores were not significantly different between treatment groups (Table 3).

**Table 3:** Histological scores at 1-year graft surveillance biopsies in the study group considered as a whole (Overall) and in the 2 treatment groups.

	Overall *(n = 110)*	AZA *(n = 55)*	MMF *(n = 55)*	Mean Diff 95% CI	*P* value
Interstitial fibrosis	0.60 ± 0.64	0.60 ± 0.60	0.60 ± 0.69	0.00 (−0.24 to 0.24)	0.9520
Tubular atrophy	0.53 ± 0.61	0.58 ± 0.61	0.49 ± 0.61	0.09 (−0.14 to 0.32)	0.4677
Allograft glomerulopathy	0.02 ± 0.14	0.02 ± 0.14	0.02 ± 0.14	0.00 (−0.05 to 0.05)	0.9893
Tubulitis	0.31 ± 0.64	0.27 ± 0.60	0.36 ± 0.68	−0.09 (−0.15 to 0.33)	0.4777
Mesangial matrix increase	0.45 ± 0.66	0.56 ± 0.78	0.34 ± 0.52	0.22 (−0.05 to 0.49)	0.0947
Vascular fibrous intimal thickening	0.58 ± 0.42	0.58 ± 0.70	0.58 ± 0.75	0.00 (−0.27 to 0.27)	1.0000
Tubular atrophy	0.53 ± 0.61	0.58 ± 0.61	0.49 ± 0.61	0.09 (−0.14 to 0.32)	0.4677
Interstitial inflammation	0.50 ± 0.69	0.45 ± 0.61	0.55 ± 0.33	−0.09 (−0.36 to 0.17)	0.4865
Arteritis	0.02 ± 014	0.02 ± 0.02	0.02 ± 0.2	0.00 (−0.6 to 0.6)	1.0000
Global score	1.13 ± 1.17	1.17 ± 1.13	1.09 ± 1.21	0.08 (−0.36 to 0.52)	0.7319

Data are mean ± SD or mean (95% CI).

AZA, azathioprine; CI, confidence interval; MMF, mycophenolate mofetil; SD, standard deviation.

#### Acute rejections

**Fig 3:**
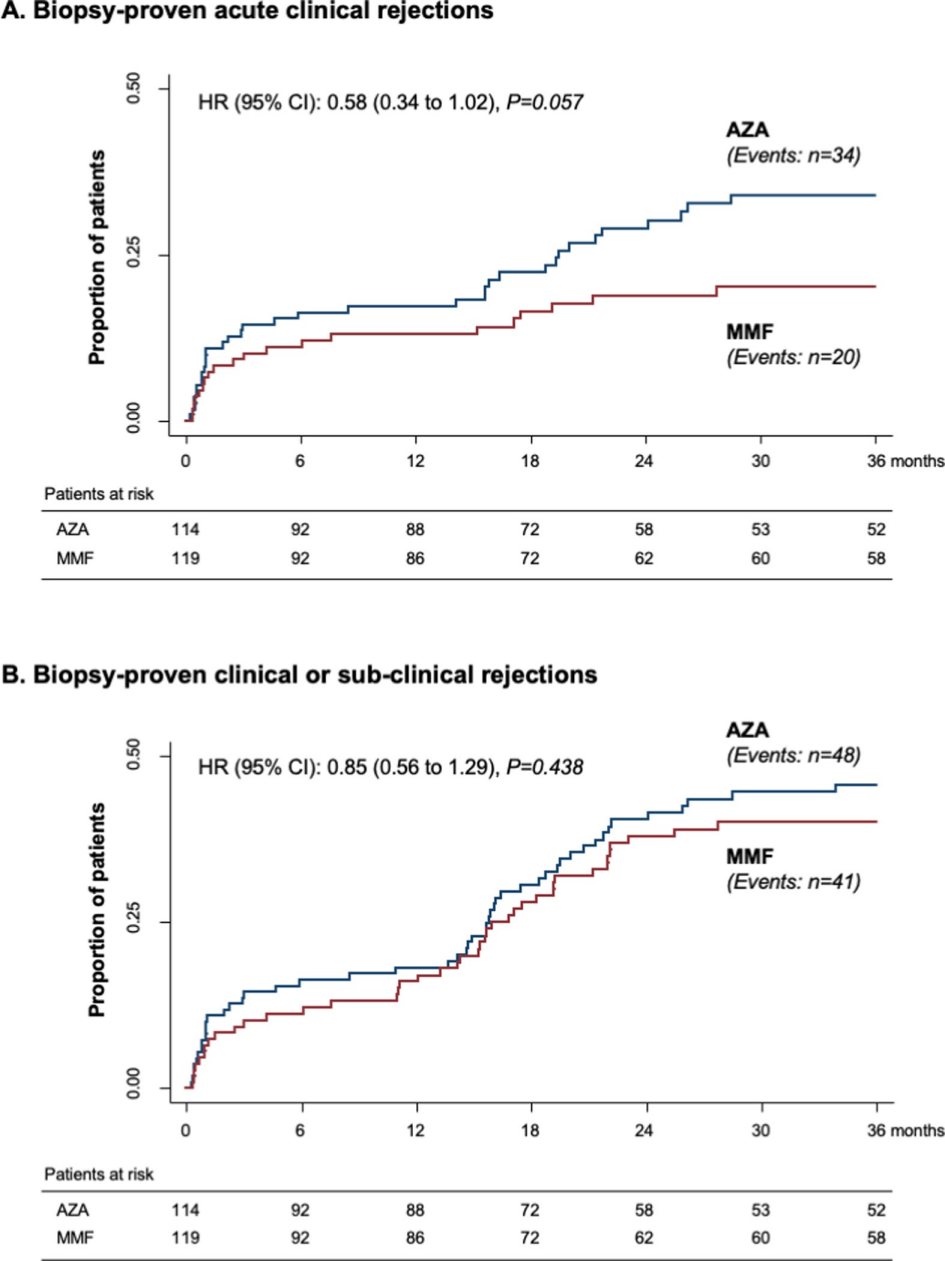
Kaplan–Meier curves of the percentages of patients with acute rejection episodes in the 2 randomization arms. Kaplan–Meier curves of the percentages of patients with biopsy-proven acute clinical rejection (**A**) or biopsy-proven clinical or subclinical rejection (**B**) during the 3 years of follow-up in the MMF (red line) and in the AZA (blue line) groups. AZA, azathioprine; CI, confidence interval; HR, hazard ratio; MMF, mycophenolate mofetil.

During the follow-up period, 54 patients had at least 1 acute rejection documented at diagnostic biopsies performed because of clinical suspicion of acute allograft rejection. Acute rejections were biopsy proven in 20 patients on MMF (16.8%) and 34 on AZA (29.8%). Despite the trend to less events on MMF, the cumulative incidence of biopsy-proven acute rejections did not differ significantly [HR (95% CI): 0.58 (0.34 to 1.02); *p* = 0.057) between treatment groups (Fig 3A and S2 Table). In 35 additional cases, histological changes consistent with the diagnosis of subclinical graft rejection were observed at surveillance biopsies from 21 patients on MMF (17.6%) and 14 on AZA (12.3%). Again, despite the trend to more events on MMF, the event rate did not differ significantly [HR (95% CI): 1.49 (0.76 to 2.92); *p* = 0.249] (S2 Table). Overall, there were 41 patients on MMF (34.5%) and 48 on AZA (42.1%) with biopsy-proven clinical or subclinical rejections, and the overall cumulative incidence of the combined event was very much the same [HR (95% CI): 0.85 (0.56 to 1.29); *p* = 0.438] between treatment groups (Fig 3B and S2 Table). In 1 patient per treatment group, acute rejection was antibody mediated. One additional patient in the AZA group had a combined cellular and vascular rejection. All T cell–mediated rejection episodes were successfully treated with steroid pulses.

#### Patient and graft survival

During the follow-up period, 9 patients (3 on MMF and 6 on AZA; *p* = 0.2776) died with a functioning graft because of cancer (1 on MMF and 3 on AZA), cardiovascular events (2 per treatment group), or infection (1 on AZA). Eleven patients, 5 on MMF and 6 on AZA (*p* = 0.7025), resumed dialysis because of graft loss. Grafts were lost because of primary nonfunction in 2 patients on MMF, progressive functional exhaustion after an acute rejection in 3 patients on MMF and 5 on AZA, and chronic rejection in 1 patient on AZA. Cox model did not show any difference between treatment groups for patient or graft survival even when adjusted for demographic and clinical covariates and cold ischemia time.

### Renal function recovery

**Fig 4:**
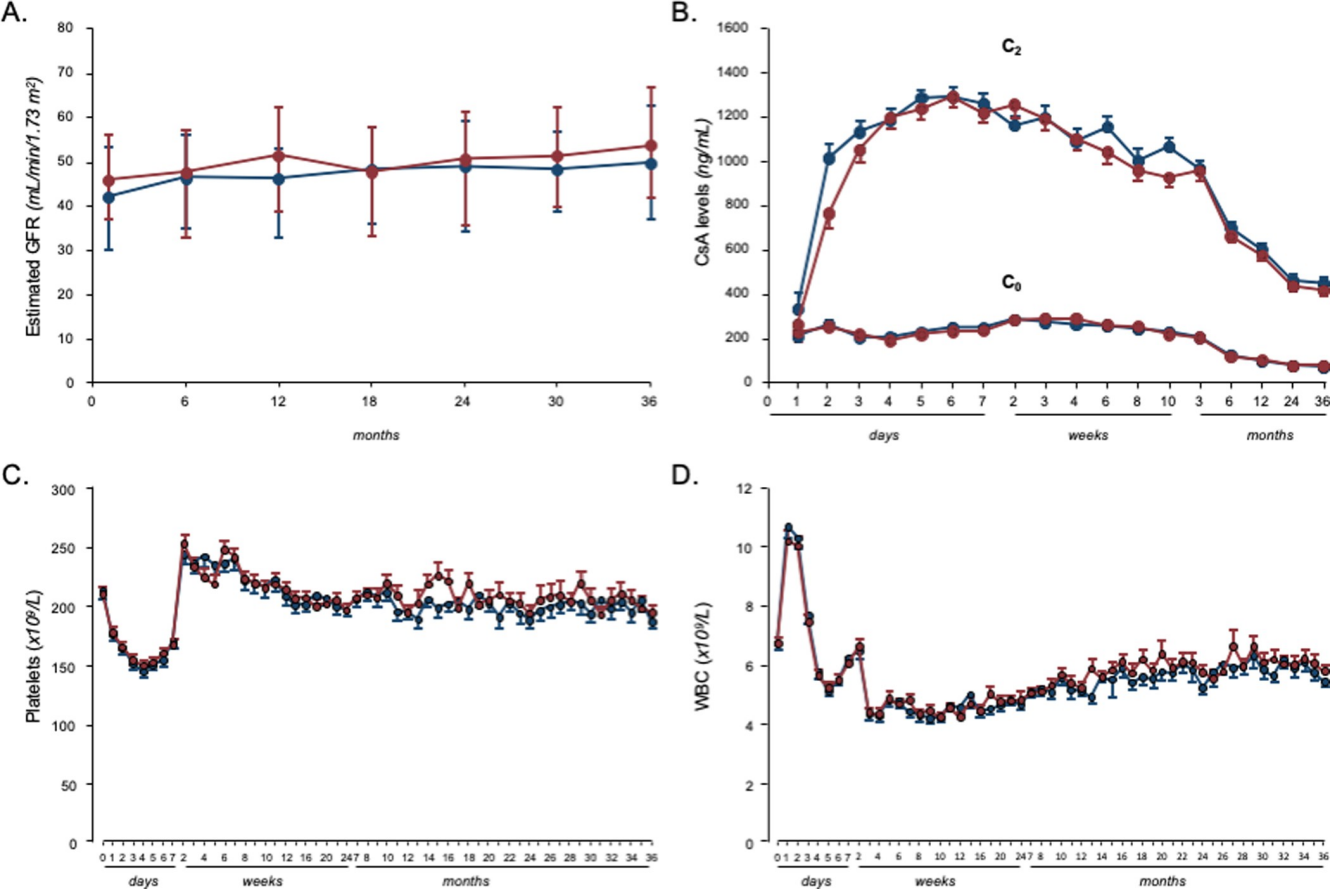
GFR, blood CsA levels, platelets, and WBC counts at different time points after transplantation in the 2 randomization arms. (A) GFR, (B) blood CsA C_0_ and C_2_ levels, (C) platelets, and (D) WBC counts at different time points after transplantation in the MMF (red) and in the AZA groups (blue). GFR data are reported as median and IQR; blood CsA levels, platelets, and WBC counts are reported as mean ± SEM. AZA, azathioprine; CsA, cyclosporine; GFR, glomerular filtration rate; IQR, interquartile range; MMF, mycophenolate mofetil; WBC, white blood cell.

Delayed graft function (DGF) was observed in 31 participants of 233 (13.3%): 14 were on MMF (11.8%) and 17 on AZA (14.9%). Event distribution was not significantly different between groups (*p* = 0.4795). Serum creatinine levels (Table 4) and eGFR were stable and not significantly different between the 2 treatment groups at each considered time point after transplant (Fig 4A).

**Table 4:** Blood pressure and main laboratory parameters at different time points after randomization in the 2 study arms.

*Months after transplant*	1	6	12	18	24	30	36
***AZA***							
***24-hour blood pressure***							
Systolic, *mm Hg*	134.9 ± 16.8	138.4 ± 15.0**	139.9 ± 16.5**	135.0 ± 17.2	134.1 ± 15.9	136.5 ± 17.4	135.3 ± 15.3
Diastolic, *mm Hg*	79.3 ± 10.2	83.4 ± 9.9**	82.8 ± 10.0**	78.5 ± 10.5	81.3 ± 8.3*	78.3 ± 8.8	80.3 ± 9.2
***Lipids***							
Total cholesterol, *mg/dL*	224.3 ± 49.5	219.5 ± 46.9	214.5 ± 46.4	211.8 ± 45.2	215.5 ± 44.6	200.6 ± 48.4	213.8 ± 42.4
LDL cholesterol, *mg/dL*	129.8 ± 33.2	136.1 ± 36.0	132.8 ± 37.1	130.3 ± 31.5	136.2 ± 35.0	118.3 ± 36.2	127.8 ± 33.5
HDL cholesterol, *mg/dL*	40.6 ± 14.6	47.6 ± 12.9**	47.1 ± 14.3**	49.4 ± 15.6**	47.8 ± 13.1**	51.7 ± 14.3**	53.0 ± 18.6**
Triglycerides, *mg/dL*	220.8 ± 101.0	167.4 ± 94.4**	152.7 ± 78.8**	147.3 ± 55.0**	148.5 ± 77.2**	152.7 ± 94.6**	146.5 ± 73.5**
***Kidney function***							
S. Creatinine, *mg/dL*	2.0 ± 1.3	1.7 ± 0.7	1.6 ± 0.8	1.5 ± 0.5**	1.5 ± 0.6*	1.6 ± 0.6	1.5 ± 0.5*
Estimated GFR, *mL/min/1*.*73m*^*2*^	42.1 [30.5; 53.8]	46.5 [36.6; 57.8]**	46.4 [39.6; 59.8]**	48.4 [38.8; 60.5]**	49.0 [38.6; 63.4]**	48.4 [39.9; 57.9]**	49.8 [36.8; 62.5]**
Proteinuria, *g/24 hours*	0.23 [0.14; 0.46]	0.19 [0.11; 0.35]	0.19 [0.12; 0.40]	-	0.16 [0.10; 0.38]	0.19 [0.14; 0.42]	0.16 [0.10; 0.28]
Albuminuria, *μg/minute*	-	15.0 [8.0; 34.0]	17.0 [6.0; 43.0]	-	16.5 [7.0; 59.0]	-	14.0 [5.0; 36.0]
***MMF***							
***24-hour blood pressure***							
Systolic, *mm Hg*	131.1 ± 17.8	138.1 ± 15.8**	136.8 ± 17.9**	129.7 ± 15.8	135.6 ± 14.6*	134.4 ± 16.5	135.5 ± 16.1*
Diastolic, *mm Hg*	77.3 ± 11.2	81.3 ± 10.6**	80.8 ± 9.3**	76.4 ± 9.8	80.2 ± 7.6**	78.9 ± 9.3	80.1 ± 9.4*
***Lipids***							
Total cholesterol, *mg/dL*	226.5 ± 52.0	215.1 ± 47.1	215.0 ± 52.3	211.4 ± 51.3	208.1 ± 42.8**	208.8 ± 40.9**	208.5 ± 40.9**
LDL cholesterol, *mg/dL*	143.6 ± 42.3	134.6 ± 37.8*	135.2 ± 42.5	124.7 ± 39.6*	129.5 ± 31.6	125.2 ± 29.1*	128.2 ± 35.0
HDL cholesterol, *mg/dL*	42.1 ± 14.5	45.3 ± 13.4**	46.2 ± 14.1**	50.4 ± 15.2**	49.1 ± 16.1**	53.2 ± 15.5**	53.2 ± 17.0**
Triglycerides, *mg/dL*	221.1 ± 93.6	166.1 ± 77.8**	152.2 ± 82.7**	164.6 ± 72.9**	146.5 ± 75.0**	145.7 ± 76.7**	147.3 ± 83.3**
***Kidney function***							
S. Creatinine, *mg/dL*	1.76 ± 1.0	1.56 ± 0.6*	1.47 ± 0.5**	1.58 ± 0.6	1.49 ± 0.5*	1.59 ± 0.9	1.46 ± 0.6**
Estimated GFR, *mL/min/1*.*73m*^*2*^	45.9 [35.5; 54.6]	47.8 [38.3; 62.6]**	51.6 [40.6; 64.2]**	47.9 [37.6; 62.6]**	50.7 [39.9; 65.5]**	51.5 [40.4; 63.2]**	53.8 [40.6; 65.7]**
Proteinuria, *g/24 hours*	0.20 [0.12; 0.39]	0.19 [0.12; 0.30]	0.18 [0.09; 0.28]	-	0.15 [0.10; 0.27]	0.20 [0.12; 0.26]	0.15 [0.11; 0.29]
Albuminuria, *μg/minute*	-	20.0 [8.0; 68.0]	16.0 [7.0; 55.0]	-	19.0 [8.0; 57.0]	-	20.5 [7.0; 61.5]

Data are means ± SD or median [IQR]. *t* Test or signed rank:

*p < 0.05,

**p < 0.01 vs month 1. All between-group comparisons statistically not significant by ANCOVA. GFR was estimated based on MDRD formula.

ANCOVA, analysis of covariance; AZA, azathioprine; GFR, glomerular filtration rate; IQR, interquartile range; MMF, mycophenolate mofetil; SD, standard deviation.

### Blood pressure, lipid, and glucose control

Systolic and diastolic blood pressure, cholesterol, and triglyceride levels were not significantly different between the 2 randomization groups during the entire follow-up period (Table 4). Eleven patients developed new-onset diabetes after transplantation (NODAT), 6 were on MMF and 5 on AZA (*p* = 0.81).

### Immunosuppressive therapy and concomitant medications

The average daily dosages of MMF and AZA at 3 years were 1,286.6 ± 228.8 mg and 80.2 ± 19.8 mg, respectively. Independent of dose tapering, CsA blood levels measured immediately before CsA morning administration (C_0_ or “trough” level) or 2 hours later (C_2_) were always not significantly different between treatment groups at baseline and at any considered time point after transplant (Fig 4B). Throughout the study period, the distribution of concomitant medications was similar between treatment groups. In particular, the proportions of patients who received at least once an angiotensin-converting enzyme inhibitor, an angiotensin II receptor antagonist, or an HMgCoA reductase inhibitor (statin) were similar in the MMF and AZA groups, whereas the proportion of patients treated with antiplatelet agents was significantly higher in the AZA group (S1 Table).

### Safety

Overall, 228 patients (98%) had at least 1 AE during the follow-up period, 115 (97%) in the MMF and 113 (99%) in the AZA group. SAEs were reported in 190 patients (82%): 96 on MMF (81%) and 94 on AZA (82%), respectively (Table 5). Seventy-five of 1,602 (4.7%) AEs and 15 of 474 (3.2%) SAEs were considered by the investigators to be related to the study drugs. The incidence of these events was not significantly different between treatment groups (Table 5). Overall, 73 patients (31%) withdrew the study drugs because of AEs, 22 patients were on MMF (18%) and 51 on AZA (45%; *p* < 0.0001). Intensity of non-SAEs was mild, moderate, or severe in 79%, 18%, or 3% of the cases, respectively, without differences between treatment groups. The total number of infections was not significantly different in the 2 study groups, with no significant differences in the incidence of cytomegalovirus reactivations.

None of the patients on AZA reported skin rash or irregular cutaneous *pigmentation*.

Despite similar trends in platelet and WBC counts between the 2 groups (Fig 4C and 4D), more episodes of nonserious thrombocytopenia or leukopenia were reported with AZA than with MMF. The number of cardiovascular events and tumors during the whole follow-up period was not significantly different in the 2 treatment arms (Table 5).

**Table 5:** Number of AEs occurring for the first time in single patients in the study group considered as a whole (Overall) and according to treatment arm.

Classification	Overall (*n* = 233)	AZA (*n =* 114)	MMF (*n* = 119)
**SAEs**	**190**	**94**	**96**
*Acute rejection*	105	51	54
*Anemia*	4	2	2
*Cardiac and vascular*	37	17	20
*DGF*	12	7	5
*Disease recurrence*	3	1	2
*Endocrin*	4	1	3
*Graft dysfunction*	22	9	13
*Hematologic*	5	4	1
*Infectious*	57	34	23
*Leukopenia*	8	3	5
*Liver and gastrointestinal*	38	17	21
*Metabolic*	4	2	2
*Mood disorder*	3	2	1
*Neoplasm bladder*	1	1	-
*Neoplasm stomach*	1	1	-
*Neoplasm hematologic*	3	2	1
*Neoplasm kidney*	8	6	2
*Neoplasm prostate*	1	1	-
*Neoplasm skin*	5	1	4
*Neurologic*	3	1	2
*Ocular*	1	1	-
*Oral*	5	2	3
*Respiratory*	28	18	10
*Skin*, *subcutaneous*, *muscuskeletal*, *and trauma*	8	5	3
*Surgical*	19	10	9
*Thrombocytopenia*	1	-	1
*Urinary*	1	1	-
*Urological*	42	21	21
*Other*	45	27	18
**Non-SAEs**	**226**	**113**	**114**
*Acute rejection*	9	7	2
*Allergy*	2	1	1
*Anemia*	100	55	45
*Cardiac and vascular*	98	48	50
*Chronic rejection*	51	20	31
*DGF*	19	10	9
*Disease recurrence*	3	3	-
*Endocrin*	41	19	22
*Graft dysfunction*	52	24	28
*Graft failure*	9	6	3
*Hematologic*	42	22	20
*Infectious*	164	80	84
*Leukopenia*	114	68**	46
*Liver and gastrointestinal*	120	59	61
*Metabolic*	197	97	100
*Mood disorder*	44	20	24
*Neoplasm eye*	1	-	1
*Neoplasm skin*	1	-	1
*Neoplasm lung*	2	1	1
*Neoplasm thyroid*	1	1	-
*Neurologic*	50	25	25
*Ocular*	31	17	14
*Oral*	24	10	14
*Respiratory*	65	34	31
*Skin*, *subcutaneous*, *musculoskeletal*, *and trauma*	74	38	36
*Surgical*	20	10	10
*Thrombocytopenia*	37	24*	13
*Urinary*	7	5	2
*Urological*	72	30	42
*Other*	152	78	74

Chi-squared or Fisher exact test vs MMF:

*p < 0.05,

**p < 0.01.

AE, adverse event; AZA, azathioprine; DGF, delayed graft function; MMF, mycophenolate mofetil; SAE, serious AE.

## Discussion

This randomized clinical trial compared the effects of MMF and AZA in 233 recipients of a first kidney graft from a deceased donor. Regardless of treatment allocation, study participants received dual induction therapy with low-dose RATG and basiliximab and steroid-free maintenance immunosuppression with lower than standard doses of CsA. During a median follow-up period of nearly 4 years, the cumulative incidence of biopsy-proven CAN (primary endpoint) in the 119 participants assigned to MMF therapy and in the 114 allocated to AZA was not significantly different. Consistently, chronic histological changes at surveillance kidney graft biopsies obtained from a subgroup of randomized participants 12 to 18 months after transplantation were also similar between treatment arms.

Donor and recipient characteristics at the time of transplant, including histological changes at pretransplant kidney graft biopsies, and the proportion of patients allocated to single or dual kidney transplant were similar between treatment groups. Blood pressure control, CsA blood levels, and the distribution of concomitant medications were also very similar between groups. Thus, observed outcomes were unlikely confounded by unbalanced distribution of risk factors between treatment arms and converged to indicate that MMF does not offer any benefit over AZA in the prevention of CAN in kidney graft recipients, even in the context of minimized maintenance immunosuppression. Finding that biopsy-proven clinical and subclinical graft rejections, patient and graft survival, DGF, and renal function recovery were also similar between treatment groups as well as the rate of AEs, in particular of those that were treatment related, provided additional evidence that the risk/benefit profile of MMF and AZA was the same, at least in our experimental setting.

Notably, subclinical rejections at prespecified surveillance biopsies tended to be more frequent in the MMF group, whereas biopsy-proven clinical rejections tended to be more frequently observed in the AZA group. Observed differences, however, were not significant, and the overall incidence of the combined endpoint of biopsy-proven clinical or subclinical rejection was remarkably similar between treatment arms. These findings provided additional evidence that MMF offered no benefits over AZA.

Regardless of treatment allocation, the rates of subclinical rejections observed in the ATHENA trial following dual induction with low-dose RATG and basiliximab were similar or lower as compared to the rates reported in previous trials using standard immunosuppression [29]. Clinical rejections were not uncommon. However, these data should be considered at the light of the reduced maintenance immunosuppression. Indeed, acute rejections in our study were less frequent than in previous trials using newly developed co-stimulatory blockers to avoid calcineurin inhibitors [30,31] or other strategies to minimize immunosuppression [32,33]. We speculate that these findings could be explained, at least in part, by the pro-tolerogenic effects of dual induction. Initial studies in nonhuman primates showed that profound T cell depletion before transplantation induces a state of donor specific immune hyporesponsiveness or even tolerance [34–36]. After depletion, graft-specific T cells regenerate slowly, so that they may become more sensitive to immune regulatory processes during their encounter with donor antigens. The additional benefits of basiliximab could be mediated by prolonged depletion of circulating B cells and prevention of their antigen-presenting activity [37]. These mechanisms could explain why previous studies indicate that combined induction with low-dose RATG and basiliximab is more effective and safer than full-dose or low-dose RATG single induction [24], especially when RATG infusion was started before graft anastomosis [25]. Study findings also confirm and extend previous evidence from MYSS [5] and MYSS follow-up [6] studies that MMF and AZA are similarly effective in preventing acute and chronic rejection in combination with standard doses of CsA microemulsion and challenge perceived superiority of MMF, largely based on industry-sponsored registration trials including patients on background immunosuppression with oil-based CsA.

The number of AEs was also not significantly different between the 2 treatment groups, including cardiovascular diseases and tumors. Notably, treatment-related leukopenia and thrombocytopenia tended to be more frequently reported in the AZA arm despite evidence of similar cell counts over time and an identical incidence of WBC counts less than 3,500/mm^3^ in the 2 treatment groups. Consistently, the percentage of patients who switched from AZA to MMF exceeded by more than 3-fold higher than the percentage of those who switched from MMF to AZA. This finding conceivably reflects the diffuse perception among physicians that MMF is devoid of bone marrow toxicity, a potentially major advantage over AZA that is emphasized by the manufacturers, but that has been challenged also in previous academic trials [5,6].

Despite the similar risk/benefit profile, AZA is approximately 15 times less expensive than MMF, and our present and previous data [5], together with studies by others [38,39], collectively support a higher cost/effectiveness of AZA over branded MMF, even in a context of minimized immunosuppression. Generic formulations for MMF are now available, but their costs also largely exceed the costs of AZA. Use of generic formulations for drugs with a narrow therapeutic index, such as immunosuppressants, is questionable [40], especially in considering that bioequivalence across generic formulations is unclear [41]. As a result, the use of branded immunosuppressive agents is still common, making our results relevant for payers and healthcare providers.

Regardless of treatment randomization, the immunosuppressive regimen we tested in ATHENA was remarkably well tolerated when compared to other regimens including standard doses of RATG or CsA [42]. The risk of infections or lymphoproliferative disorders was lower when compared to standard triple immunosuppressive regimens without induction therapy [43], a risk that can be further increased by novel immunosuppressants introduced in clinics to replace calcineurin inhibitors [44]. Posttransplant new-onset diabetes was reported in only 11 of 233 randomized study participants, an event rate that is remarkably lower as compared to rates reported in previous studies with regular doses of calcineurin inhibitors or with chronic use of steroids. This finding may have major clinical implications, because up to 40% of kidney transplant recipients can develop diabetes by the third year posttransplantation [45] and posttransplant diabetes is a major risk factor for cardiovascular events and even graft rejection [46].

### Study limitations and mitigating factors

The relatively small sample size and lack of blinding were major limitations of the study. These limitations are largely explained by resource constraints related to the fully academic nature of the study, which was partially sponsored by the Italian Medicines Agency (AIFA), without company support. The open design also reflected the philosophy of AIFA that preferentially sponsored pragmatic trials expected to generate results directly generalizable to every day clinical practice.

We prespecified that CAN was the primary endpoint of the study because, when the trial was designed, CAN was the most widely used score of chronicity [28,47]. Since study initiation, the use of other chronicity scores has been progressively suggested to better describe chronic allograft injury [48].

Because of clinical judgment of investigators in charge of study participants, dictated in particular by concerns about safety and futility of the procedure, in some cases surveillance biopsies were not performed despite specific per-protocol indications. The decision to deviate from the study protocol was discussed with the first investigator and generally was approved in respect of the pragmatic nature of the study. This approach unlikely introduced a bias in data analyses because reasons for deviations from study protocols were similar between groups. Moreover, the incidence of CAN was so close with MMF or AZA, that a potential difference between groups was very unlikely missed because of the lower than expected number of performed per-protocol biopsies.

Finally, lack of standardized measurement of donor-specific antibodies (DSAs), a major risk factor for graft survival [49], is another limitation of the study, especially in consideration of the relatively short follow-up period. Future studies are needed to assess the difference between AZA and MMF in preventing the development of DSA.

The purely academic nature of the study is a major strength, because no company involvement in any aspect of study conduct protected the trial from the risk of bias in favor of sponsors’ products [50] and reinforced the integrity of study findings. Protocol guidelines to patients’ treatment and monitoring reflected standard practice in many transplant centers, which enhanced the generalizability of the study findings to real world. Serial per-protocol histological evaluations at the time of transplant and during the follow-up period strengthened the robustness of the findings. The use of CAN as a surrogate for long-term graft outcomes is supported by a strong association between development of CAN and long-term graft failure [51,52], which corroborates the robustness and relevance of our findings.

## Conclusions

In conclusion, this randomized clinical trial comparing the risk/benefit profile of MMF and AZA in recipients of a first kidney transplant from a deceased donor found that, in the setting of a steroid-free immunosuppressive regimen with low-dose maintenance CsA, the rate of CAN, biopsy-proven clinical and subclinical rejections, patient and graft survival, renal function recovery, and AEs were similar between treatment groups. Although the trial was powered on a relatively large effect size and the CIs for the primary endpoint do not allow to formally rule out differences between groups, the 2 treatment arms yielded virtually identical results in terms of efficacy and safety, making this hypothesis unlikely.

Our findings may have implications for healthcare providers because MMF, even in its generic formulations, is an expensive medication, and the preferential use of AZA instead of MMF for maintenance immunosuppression would remarkably reduce the costs of kidney transplantation, without affecting graft outcomes. Considering also the reduced costs for low-dose CsA chronic therapy, this strategy might facilitate the access to the procedure in particular in resource-limited settings. Our data may also serve to prove the concept that maintenance immunosuppression can be minimized in carefully selected patients to avoid the side effects of steroids and limit the nephrotoxicity of calcineurin inhibitors without the excess costs of novel expensive immunosuppressants. Whether our findings can be extended to recipients receiving other forms of induction or maintenance immunosuppression, a second kidney graft or those at higher immunological risk of rejection is worth investigating.

## Supporting information

S1 Text ATHENA study organization. (DOCX)

S2 Text Study protocol and amendments. (PDF)

S3 Text CONSORT checklist. (DOC)

S4 Text Ethics approval document. (PDF)

S1 Table Concomitant medications throughout the study period. (DOCX)

S2 Table Acute rejections in the study group considered as a whole (Overall) and in the two treatment groups. (DOCX)

## Abbreviations

ACR: acute cellular rejection
AE: adverse event
ANCOVA: analysis of covariance
ATHENA: A randomized, prospective, multicentre trial to compare THe Effect on chronic allograft Nephropathy prevention of mycophenolate mofetil versus Azathioprine
AZA: azathioprine
CADI: chronic allograft damage index
CAN: chronic allograft nephropathy
CI: confidence interval
CONSORT: Consolidated Standards of Reporting Trial
CRC: Clinical Research Center
CsA: cyclosporine
DGF: delayed graft function
DSA: donor-specific antibody
eGFR: estimated GFR
ESKD: end-stage kidney disease
GFR: glomerular filtration rate
HR: hazard ratio
IL-2: interleukin 2
IQR: interquartile range
MMF: mycophenolate mofetil
MYSS: MYcofenolate Steroid Sparing
NODAT: new-onset diabetes after transplantation
RATG: rabbit thymoglobulin
SAE: serious AE
SD: standard deviation
WBC: white blood cell
