# Supplementary material for: Mycophenolate mofetil versus azathioprine in kidney transplant recipients on steroid-free, low-dose cyclosporine immunosuppression (ATHENA): A pragmatic randomized trial
Source: PLoS Med. 2021 Jun 24;18(6):e1003668. doi: 10.1371/journal.pmed.1003668 (PMC8224852; doi:10.1371/journal.pmed.1003668)
Supplement: S1 Text — (DOCX) [file pmed.1003668.s001.docx]

**ATHENA Study Organization**

***Coordinating Centre:*** Mario Negri Institute for Pharmacological Research IRCCS, Clinical Research Centre for Rare Diseases Aldo e Cele Daccò, Villa Camozzi, Ranica (Bergamo); Scientific Responsible: Norberto Perico (Ranica) Study coordinator: Giuseppe Remuzzi (Bergamo).

***Centres including patients:*** Unità Operativa di Nefrologia e Dialisi - Dipartimento di Immunologia e Clinica dei Trapianti, ASST Papa Giovanni XXIII, Bergamo (Giuseppe Remuzzi, Piero Ruggenenti, Eliana Gotti, Annarita Plati, Ettore Sabadini, Maddalena Marasà, Alessia Gennarini, Valentina Portalupi, Stefano Rota, Giorgio Gentile) and Clinical Research Centre for Rare Diseases Aldo e Cele Daccò, Villa Camozzi (Norberto Perico, Matias Trillini, Silvia Prandini, Veruska Lecchi, Monica Cortinovis, Giulia Gherardi, Daniela Cugini), n = 130; Divisione di Nefrologia e Dialisi - Azienda Ospedaliera Spedali Civili di Brescia, (Silvio Sandrini, Nicola Bossini, Raffaella Chiappini, Gisella Setti, Francesca Valerio, Regina Tardanico), n = 52; Dipartimento di Scienze Chirurgiche, Policlinico Gemelli Roma (Franco Citterio, Antonio Gargiulo, Gionata Spagnoletti, Josè Alberto Pedroso, Evaldo Favi, Maria Paola Salerno, Jacopo Romagnoli, Valentina Pietroni, Alessia Toscano), n = 24; Unità di Nefrologia dei Trapianti - Azienda Ospedaliera Ospedale Niguarda Cà Granda Milano (Giacomo Colussi, Enrico Minetti, Alessia Comino, Maria Chiara Sghirlanzoni), n = 16; Seconda Università degli Studi di Padova – Ospedale Giustinianeo Istituto di Chirurgia Generale Padova (Paolo Rigotti, Manuela Lazzarin, Lucrezia Furian), n = 7; Unità di Nefrologia e Emodialisi - Azienda Ospedaliero-Universitaria S. Maria della Misericordia Udine (Domenico Montanaro, Maria Groppuzzo, Patrizia Tulissi), n = 4.

***Centres not including patients:*** Divisione di Nefrologia - Istituto Italiano per i Trapianti e Terapie ad Alta Specializzazione ISMETT Palermo (Bruno Gridelli, Tullio Bertani), Unità Operativa Nefrologia e Dialisi - Ospedale Regionale di Circolo e Fondazione Macchi Varese (Donato Donati)

***Activities of the Coordinating Clinical Research Centre:*** Monitoring, Drug Distribution and Pharmacovigilance (Nadia Rubis, Wally Calini, Olimpia Diadei, Alessandro Villa, Davide Villa); Database and Data Validation (Davide Martinetti, Sergio Carminati); Randomisation (Giovanni Antonio Giuliano); Data Analysis (Annalisa Perna, Francesco Peraro); Centralised Laboratory Measurements (Flavio Gaspari, Silvia Ferrari, Nadia Stucchi, Antonio Nicola Cannata); Regulatory Affairs (Paola Boccardo, Sara Peracchi).
