# Supplementary material for: Mycophenolate mofetil versus azathioprine in kidney transplant recipients on steroid-free, low-dose cyclosporine immunosuppression (ATHENA): A pragmatic randomized trial
Source: PLoS Med. 2021 Jun 24;18(6):e1003668. doi: 10.1371/journal.pmed.1003668 (PMC8224852; doi:10.1371/journal.pmed.1003668)
Supplement: S2 Text — (PDF) [file pmed.1003668.s002.pdf]

ISTITUTO DI RICERCHE FARMACOLOGICHE **MARIO NEGRI**

Centro di Ricerche Cliniche per le Malattie Rare "Aldo e Cele Daccò"

Villa Camozzi – 24020 Ranica (BG) – Italy, tel +39.035.45351 - fax +39.035.4535.371
villacamozzi@marionegri.it – http://villacamozzi.marionegri.it

A RANDOMIZED, PROSPECTIVE, MULTICENTER TRIAL TO COMPARE THE EFFECT ON CHRONIC ALLOGRAFT NEPHROPATHY PREVENTION OF MYCOPHENOLATE MOFETIL VERSUS AZATHIOPRINE AS THE SOLE IMMUNOSUPPRESSIVE THERAPY FOR KIDNEY TRANSPLANT RECIPIENTS (ATHENA Study)

Running title

MMF vs. AZA for kidney transplantation

Key words: Mycophenolate mofetil, azathioprine, kidney transplant, chronic allograft nephropathy

Prepared by

Piero Ruggenenti, Paolo Cravedi, Norberto Perico, Giuseppe Remuzzi

Revised and approved by:

Donato Donati, Bruno Gridelli, Piergiorgio Messa, Paolo Rigotti, Maurizio Salvadori, Silvio Sandrini

Final version

Ranica, June 3, 2008

Amended 5

Bergamo, September 30th, 2011

I CONTRIBUTI PER LA RICERCA VERSATI ALL'ISTITUTO SONO FISCALMENTE DEDUCIBILI DAL REDDITO (Gazzetta Uff. N.179 del 3/8/2011)
FONDAZIONE PER RICERCHE ERETTA IN ENTE MORALE, D.P.R. 361 DEL 5/4/1961 - REGISTRO PERSONE GIURIDICHE PREFETTURA MILANO N.227
CONTO CORRENTE POST. N.58337205 - COD. FISC. E PARTITA IVA 03254210150 - ANAGRAFE NAZIONALE RICERCHE COD.G1690099
RECOGNIZED AS A TAX EXEMPT ORGANIZATION UNDER SECTION 501 (c)(3) OF THE USA INTERNAL REVENUE CODE-TAX I.D. No.: 98-6000957
Sistema di gestione qualità certificato da Certiquality UNI EN ISO 9001:2008,
progettazione ed erogazione di corsi di formazione specialistica nell'ambito della biologia e della medicina
SEDE LEGALE: Via Giuseppe La Masa, 19 - 20156 Milano MI - Italy

Confidential Statement

This protocol contains strictly confidential information which is not to be communicated or published unless previously authorized by

Mario Negri Institute for Pharmacological Research

Clinical Research Center for Rare Diseases

SUMMARY

Background - Steroid-free, low-dose cyclosporine (CsA) and mycophenolate mofetil (MMF) therapy combined to induction with basiliximab and low-dose Rabbit Anti-human Thymocyte Globulin (RATG) effectively prevented acute rejection in kidney transplant recipients. Steroid-free therapy with CsA and MMF has been also associated with a reduced risk of chronic allograft nephropathy (CAN), a frequent cause of late graft loss. Whether basiliximab/low-dose RATG induction plus MMF monotherapy may prevents CAN even in a CsA-free regimen, without increasing the risk of acute rejection, is worth investigating. The MYSS trial found that MMF and AZA are equally effective in preventing acute rejection after steroid withdrawal. Whether AZA might also be as effective as MMF in preventing CAN in a CsA-free immunosuppressive regimen is unknown.

Objectives - The study will primarily compare the incidence of biopsy-proven CAN three years post-transplant in kidney recipients randomly allocated to MMF or AZA, after induction therapy with basiliximab and low-dose RATG, and sequential steroid and CsA withdrawal. Secondarily, the study will compare acute rejections after CsA withdrawal, long-term patient and graft survival, and graft function and prevalence/severity of CAN at study end.

Methods - Two-hundred-twenty-four kidney transplant recipients from deceased donors given induction therapy with two 20 mg basiliximab injections 4 days apart and a seven-day course of RATG (0.5 mg/kg/day), will be randomly allocated on a 1:1 basis to 3-year treatment with low-dose MMF or AZA, added-on CsA maintenance therapy. At 1 year, rejection-free patients with no evidence of tubulitis at kidney biopsy will withdraw CsA and will have a kidney biopsy 3 year post-transplant for evaluating the presence and severity of CAN. Should the cumulative incidence of acute rejection exceed 15% during CsA withdrawal the study will be stopped. Should the incidence differ by >30% between the two treatment arms, all patients will be given the most effective treatment and the follow up will be continued. A final biopsy will be repeated 4 years post-transplant.

Expected results - Most patients are expected to be effectively maintained on single drug immunosuppression, which implies less steroid- and CsA- related complications and treatment costs. MMF is expected to prevent CAN more effectively than AZA. However, should AZA be more or as effective compared to MMF, at study end all patients could be shifted to AZA, that is 15-fold less expensive than MMF. Extended to clinical practice, these findings should translate in improved patient care and major cost-savings for the Health Care System.

BACKGROUND

The introduction of triple-therapy regimens that include a calcineurin inhibitor, steroids, and azathioprine (AZA) or mycophenolate mofetil (MMF) greatly reduced the risk of acute rejection in renal transplantation[1]. However, the long-term use of both calcineurin inhibitors and steroids is associated with serious toxicities that ultimately impact patient and graft survival[2,3]. Adverse effects associated with their chronic use include hypertension, dyslipidemia, diabetes and osteoporosis[4,5]. Moreover, nephrotoxicity associated with calcineurin inhibitors therapy is one of the major determinants of chronic allograft nephropathy (CAN), the main cause of late graft failure[6]. Minimisation of chronic immunosuppression is therefore of paramount importance to improve patient and graft survival. Thus, the quest for strategies inducing specific immune hyporesponsiveness or even tolerance – ideally via short-term interventions that would target only the pathogenic immune response and leave the protective host immune response unimpaired – has provided a “holy grail” for transplant immunologists.

In non human primates, profound T-cell depletion before allotransplantation with gradual posttransplant T-cell repopulation induces a state of donor specific immune hyporesponsiveness or even tolerance. Indeed, after depletion, graft-specific T-cells regenerate slowly, so that they may become more sensitive to immune regulatory processes during their encounter with donor antigens[7,8]. Consistently with these experimental findings, we recently found that induction therapy with basiliximab and lower (about one fourth) than conventional doses of Rabbit Anti-human Thymocyte Globulin (RATG), combined to low-dose CsA and MMF maintenance therapy, allowed early withdrawal of steroids without increasing the risk of rejection (only 6 % of patients had an acute and reversible rejection episode up to six months post-transplan) in high risk patients such as second transplant or hyperimmune recipients or recipients with delayed graft function[9]. Of note, unlike induction protocols with “standard” RATG doses, the above regimen was extremely well tolerated, avoided the risk of acute cytolysis reactions (fever, severe leukopenia or thrombocytopenia), reduced the incidence of immuno-hemolytic anemia and the need for red blood cell transfusions. Moreover, this approach did not increase the risk of CMV reactivations or lymphoproliferative disorders, even as compared to standard triple immunosuppressive regimens without induction therapy[9].

The doses of CsA employed in the above protocol were about half the doses currently used in clinical practice. However, even these very low doses have been reported to have a significant toxicity that, in the long-term, may adversely affect the graft function and survival[10]. Thus, implementing innovative immunosuppressive strategies allowing to early and safely withdraw

calcineurin inhibitor therapy might have major clinical implications in term of improved kidney function and long term survival. This would also avoid the adverse effects of chronic CsA therapy on arterial blood pressure, lipid profile and blood glucose that, altogether, remarkably increase the overall cardiovascular risk in these patients.

Previous experience is available that in patients on maintenance immunosuppressive therapy with steroids and MMF, CsA withdrawal, despite a small excess in acute rejection episodes, may result in an improvement in renal function at 1 and 5 years[11]. These patients were therefore protected from the chronic effect of CsA therapy, but continued to be exposed to those of steroids. Here we propose to test the possibility to withdraw both steroids and CsA, without unacceptably increasing the risk of acute rejection or CAN. To this purpose, induction therapy with basiliximab plus low-dose RATG might help inducing a condition of reduced immuno-responsiveness that might allow to sequentially withdraw steroids and CsA without adversely affect the outcome of the graft.

Evidence that MMF suppresses the production of anti-HLA antibodies, inhibits the recruitment of mononuclear cells into the allograft, as well as the proliferation of arterial smooth muscle cells, has been taken to suggest that MMF might play an important protective effect against the development and progression of chronic allograft nephropathy[12-16]. Thus, an immunosuppressive regimen based on low-dose MMF as sole antirejection drug not only would avoid chronic toxicity of steroids, and calcineurin inhibitors, but would also limit the risk of CAN, the main cause of allograft loss in the long-term. On the other hand, however, the Mycophenolate Steroid Sparing (MYSS) trial[17] found that AZA was as effective as MMF in preventing acute allograft rejection in CsA-treated kidney transplant recipients, even after steroid withdrawal. Since acute allograft rejection is one of the strongest predictor of CAN, these findings can be taken to suggest that, in the long-term, AZA might share with MMF also a similar protective effect against the development of CAN. Moreover, it must be emphasized that chronic CsA nephrotoxicity is a major component of CAN. Thus, the prevalence and severity of CAN may be reduced in patients on CsA-free immunosuppressive regimens. In this clinical setting, the benefits of MMF against the development of CAN might not appreciably exceed those of MMF. Should this be the case, AZA might confer the same benefits of MMF, but at remarkably lower costs since, at equivalent doses, AZA is about 15 times less expensive than MMF.

Regardless of the above, MMF or AZA monotherapy would avoid or limit most of the complications of chronic immunosuppressive regimens including steroids and calcineurin inhibitors, such as metabolic, osteomuscolar and cardiovascular diseases, cancer and opportunistic infections [18,19]. In clinical practice this approach might offer a number of advantages for the Italian National Health Service. First of all, this strategy would reduce the direct costs for the

immunosuppressive drugs that will be progressively withdrawn, a saving that will overwhelm the additional expenses for the induction therapy with basiliximab and RATG. Secondarily, this strategy will allow reducing the treatment costs for steroid- and CsA- related complications and, more in general, for complications associated with over-immunosuppression. Finally, should AZA result as effective as MMF in reventing CAN, most kidney transplant recipients could be maintained on long term monotherapy with AZA, that is with an extremely cheap drug that s available as a galenic formulation. The cost savings for the National Health Service would be quite consistent. Indeed, the MYSS trial[17] showed that if AZA is used instead of MMF, more than €4000 per patient per year are saved. With a population of 10,000 renal transplant patients in Italy, use of AZA rather than MMF should give a net yearly saving of about € 50 million.

AIMS

Primary

To compare the incidence of CAN[20] 3 years post-transplantation in patients receiving induction therapy with basiliximab and low-dose RATG and randomized to maintenance immunosuppression with low-dose MMF or AZA monotherapy.

Secondary

1 year

- To assess the overall cumulative incidence (regardless of randomization) of acute rejections and of tubulitis at 1-year histology evaluation
- To compare the cumulative incidence of acute rejections and of tubulitis in the two treatment groups

2 years

- To assess the overall cumulative incidence (regardless of randomization) of biopsy-proven acute rejection during CsA tapering
- To compare the cumulative incidence of biopsy-proven acute rejections in the two treatment groups

3 years

- To assess the overall incidence (regardless of treatment randomization) of CAN and the possible relationships between the histology changes at 3 years and the histology findings at pre-transplant (baseline) kidney evaluation or previous acute rejection episodes observed before or after CsA withdrawal

- To asses the global (vascular, glomerular and tubular-interstitial) score of chronic histology changes[21] compared to baseline in the study group as a whole, in the two treatment arms and within the two subgroups completing or not completing CsA withdrawal

4 years

- To assess patient and graft survival and function, incidence of CAN, and possible relationships between the histology changes at 4 years and the histology findings at baseline and at 3 years post-transplant, or previous acute rejection episodes observed before or after CsA withdrawal
- To compare overall patient and graft survival and function, incidence of CAN and the global histology score in the two treatment groups

PATIENTS

Patients will be identified among the subjects who are referred to the transplant centers involved in the trial and selected to receive a kidney transplant according to standardized clinical criteria.

Inclusion criteria

- Males and females aged 18 years or more;
- First single or double kidney transplant from deceased donors;
- Written informed consent.

Exclusion criteria

- Specific contraindications to RATG therapy such as severe leucopenia (WBC<2000/mm^3);
- High immunological risk – such as second transplant recipients or those who have a panel reactivity $\geq$ 10%;
- History of malignancy (except non metastatic basal or squamous cell carcinoma of the skin that has been treated successfully;
- Evidence of active hepatitis C virus, hepatitis B virus or human acquired immunodeficiency virus infection;
- Any chronic clinical conditions that may affect completion of the trial or confound data interpretation;
- Pregnancy or lactating;
- Women of childbearing potential without following a scientifically accepted form of contraception;

- Legal incapacity and/or other circumstances rendering the patient unable to understand the nature, scope and possible consequence of the trial;
- Evidence of an uncooperative attitude;
- Any evidence that patient will not be able to complete the trial follow-up.

RANDOMIZATION

Randomization will be centralized at the Laboratory of Biostatistics of the Clinical Research Center for Rare Diseases “Aldo e Cele Daccò” Villa Camozzi, Ranica, Bergamo of the Mario Negri Institute for Pharmacological Research under the responsibility of an independent investigator.

STUDY DESIGN

This will be an open, randomized, prospective, multicenter study to compare the cost/efficacy of low-dose MMF versus AZA as the sole immunosuppressive therapy in preventing CAN after induction therapy with basiliximab plus low-dose RATG combined with CsA during the first year after surgery in kidney transplant recipients. An histology evaluation of kidney graft before transplantation, although not mandatory, will be strongly recommended. At the end of the first year post-transplant (Phase A), patients with no previous biopsy-proven acute rejection and no histology evidence of tubular mononuclear infiltration (tubulitis) will progressively taper/withdraw CsA therapy. Patients with stable kidney function who at 1 year graft biopsy show interstitial infiltrates, but not evidence of tubulitis (the infiltrates may reflect a modulatory response of the host rather a sub-clinical rejection), will undergo progressive tapering/withdrawal of CsA therapy. Similarly, patients that cannot perform graft biopsy at 1 year post transplant for technical/clinical reasons, and who had no previous clinical or biopsy-proven acute rejection, will also progressively taper/withdraw CsA therapy.

(Patients with recurrence of primary renal disease on the graft may not enter the tapering/withdrawal program in the case the Investigator will consider that this might confound data interpretation. This possibility, however, will be discussed with the study co-ordinator and reasons for not tapering/withdrawing CsA will be reported in the CFR. The patient will be maintained on active follow-up). All patients with no previous acute rejection (biopsy proven or not) during the first year will have a measurement of circulating anti HLA-antibodies at 12 months after transplant. Presence of anti-HLA antibodies (either donor specific or not) will represent a contraindication to CsA tapering. The same measurements will be performed also at month 18, 24, and 30.

In order to standardize CsA tapering and achieving CsA withdrawal over an homogeneous follow-up period, patients will reduce the initial CsA dose by about 10% every 4 weeks. By this approach, 50% tapering should be achieved in 24 weeks. After additional 6 months at 50% of the initial CsA dose in addition to MMF or azathioprine as maintenance immunosuppressive therapy, should no rejection episodes occur (second year biopsy), a further progressive tapering of CsA dose will be attempted up to complete CsA withdrawal. Tapering will be scheduled as above, and the initial CsA dose (at time of 1st graft biopsy) will be reduced by about 10% every 4 weeks. Thus, complete CsA withdrawal will be achieved 18 months after starting the initial CsA tapering, i.e at 29-30 months post-transplantation.
The broad aim, however, should be to complete CsA withdrawal within 10-12 months. Thus, from month 30 post-transplant patients successfully withdrawing CsA will remain on MMF or AZA as the sole maintenance immunosuppression (Phase B).
Acute graft rejections will be initially treated with methylprednisolone pulses according to the centre's practice and rescue therapy with monoclonal antibodies or thymoglobulins will be allowed for steroid-resistant cases. After resolution of the rejection episode and recovery/stabilization of graft function, the immunosuppressive treatment preceding the dose reduction associated with the event will be restored. Minor adjustments in maintenance therapy will be allowed whenever deemed appropriate to minimize the risk of further rejection episodes. These deviations will be reported and the reasons will be described in the CRF. The immunosuppressive therapy will not be modified in those patients who will not enter the phase B, because of biopsy-proven acute rejection during the first year after transplant or histology evidence of mononuclear tubular infiltration (i.e. tubulitis) (or disease recurrence that might confound data interpretation) at the biopsy performed at 1 year post-transplant. Patients will be maintained on active follow-up.

Any acute rejection episode, as well as any other serious adverse event, will be reported within 24 hour to the coordinating center that, every month, will report all the events to the Safety Panel (see appendix). Should the cumulative incidence of acute rejections observed during the CsA tapering/withdrawal period exceed 15% (that is the incidence previously observed in the MYSS trial during steroid withdrawal[17]) the study will be closed. Should the incidence differ by more than 30% between the treatment arms (that is the difference observed between MMF and AZA in the MMF registration studies[22,23], all patients will be given the treatment associated with the lower incidence of rejections (see appendix) and the study will be continued. At 3 years after transplant, a kidney biopsy will be performed in all patients, in order to assess the presence and severity of CAN in the study group as a whole as well as in the two subgroups completing or not completing CsA

withdrawal. If one of the two study treatments (MMF or AZA) will be associated with a significantly lower incidence of CAN, all patients will be treated with the more effective drug. Patients will be maintained on active follow-up and a final biopsy will be performed at completion of the 4th year follow up in order to evaluate long-term patient and graft survival and the potential regression of graft changes and dysfunction in patients shifted from the less to the more effective drug.

Immunosuppressive protocol

- *Induction therapy.* All patients will receive induction therapy with basiliximab (two 20 mg injections: the first one pre-operatively, the second one 4 days post-transplant) plus RATG low-dose (0.5 mg/kg/day for 7 days, starting on the day of transplant). Patients will also receive intravenous methylprednisolone on day 0 (500 mg), day 1 (250 mg) and day 2 (125 mg) and oral prednisone on day 3 (75 mg), 4 (50 mg), 5 (25 mg) and 6 (25 mg). Thereafter, patients will be free of steroid therapy.
- *Cyclosporine.* CsA dose will be adjusted to maintain trough levels between 300 and 400 ng/mL during the first week after transplantation, between 200 and 250 ng/mL up to month 2, between 150 and 200 ng/ml from month 3 to month 4, and between 100-150 ng/ml thereafter. CsA will be orally administered in two divided daily doses that will be adjusted in order to achieve and maintain the above targets. Intravenous CsA infusion from the time of transplant will be allowed and will be followed by oral CsA according to center practice. Through (C0) blood levels will be monitored every week in the first month post-surgery, every 2 weeks during the month 2-4, and every month thereafter, and whenever deemed clinically appropriate.
- *Mycophenolate mofetil.* Patients randomized in this group will receive 750 mg of MMF per os twice a day starting on the day of transplant. MMF dose will be reduced in case of white blood cell count lower than $2{,}000/mm^3$ and whenever deemed clinically appropriate. Any change in MMF dose and the reason for the change will be reported in the CRF.
- *Azathioprine.* Patients randomized in this group will receive 75 mg of AZA per os (or 125 mg if body weight > 75 kg) once a day starting on the day of transplant. AZA dose will be reduced in case of white blood cell count lower than $2{,}000/mm^3$ and whenever deemed clinically appropriate. Any change in AZA dose and the reason for the change will be reported in the CRF.

CsA tapering/withdrawal

CsA dose will be reduced by about 10% of the initial dose (at 1 year) every 4 weeks. By this approach, 50% CsA tapering should be achieved in 20-24 weeks. After additional 6 months at 50% of the initial CsA dose, should no acute rejection episodes occur (second year biopsy), a further progressive tapering of CsA dose will be attempted up to complete CsA withdrawal. CsA dose will be reduced by about 10% every 4 weeks (reference dose is the initial CsA dose at the time of 1st graft biopsy).

Deviations from this schedule are allowed as deemed appropriate. They should be reported together with the reason for the deviation in the CRF. The broad aim, however, should be to complete CsA withdrawal within 18 months (i.e. at 29-30 months post transplantation). After complete CsA discontinuation, the patients will remain on monotherapy with low-dose MMF orAZA.

Patient monitoring

Serum creatinine will be monitored at least every week during the CsA tapering period and up to 2 weeks after CsA withdrawal. During the MMF/AZA monotherapy phase, serum creatinine will be monitored at monthly intervals. CsA trough levels will be monitored every months.

During CsA tapering/withdrawal or MMF/AZA monotherapy period, patients with increase in serum creatinine levels $\geq$0.3 mg/dl over the last value, will repeat the measurement. If the increased serum creatinine concentration is confirmed, and renal ultrasound findings exclude urinary tract obstruction or other complications, a renal biopsy should be performed, unless medically contraindicated. Patients will be treated according to the histology findings. If acute graft rejection will occur, methylprednisolone pulses will be administered according to the centre's practice. Rescue therapy with monoclonal antibodies or thymoglobulins will be allowed for steroid resistant rejections. After resolution of the rejection episode and recovery/stabilization of graft function, the the immunosuppressive treatment preceding the dose reduction associated with the event will be restored.

OUTCOME VARIABLES

Primary

Cumulative incidence of biopsy-proven CAN at 3 years follow-up in patients completing CsA withdrawal in the two treatment groups (end phase B).

Secondary

1 year

- Cumulative incidence (regardless of randomization) of acute rejections and of tubulitis at 1-year histology evaluation
- Cumulative incidence of acute rejections and of tubulitis in the two treatment groups

2 years

- Cumulative incidence (regardless of randomization) of biopsy-proven acute rejections during CsA tapering
- Cumulative incidence of biopsy-proven acute rejections in the two treatment groups

3 years

- Cumulative incidence (regardless of treatment randomization) of CAN in the study group as a whole and within the two subgroups completing or not completing CsA withdrawal
- Cumulative incidence of biopsy-proven CAN in patients not completing CsA withdrawal in the two treatment groups.
- Global (vascular, glomerular, and tubular-interstitial) score of chronic histology changes[21]

4 years

- Patient and graft survival and function, CAN, global histology score in the study group as a whole, in the two treatment arms and the two subgroups completing or not completing CsA withdrawal

Safety variables

They will include serious and non-serious adverse events and the clinical or laboratory parameters routinely recorded in clinical practice to monitor the tolerability of immunosuppressive therapy and concomitant medications

SAMPLE SIZE

Primary efficacy comparison will consider the incidence of CAN at 3 years post-transplant in the MMF and AZA groups. No data from randomized clinical trials on the incidence of CAN at three years are available. One study reported a 46% incidence on MMF at 1 year compared to a 71% incidence on AZA. Assuming, conservatively, a similar incidence at 3 years, to give the trial an 80% power to detect by a two-side test (□=0.047, log-rank test), the expected difference in CAN incidence, 70 patients per group should complete the study. Thus, if CsA withdrawal will be feasible in 70% of randomized patients, 100 patients per group should enter the study. To account

for the possible drop-outs, 112 patients per group should be included for a total of 224 patients. No sample size is calculated for secondary comparisons.

STATISTICAL ANALYSIS

Three different patients populations will be identified: a. the 'Full Analysis Set', b. the 'Per-Protocol Set' and c. the 'Safety Set'. The 'Full Analysis Set' will include all randomised and transplanted patients, taking at least one dose of study medication. The 'Per-protocol Set' will include all randomised and transplanted patients, taking at least one dose of study medication, excluding major protocol violators. The 'Safety Set' will include all randomised and transplanted patients, including those who will not take any dose of study medication. Randomised patients not undergoing renal transplant are not part of the population and therefore excluded from the Full Analysis Set, from the 'Per-Protocol Set' and from the Safety Set. The different exclusions from the Full Analysis Set, from the Per-Protocol Set and from the Safety Set will be described in more detail in the Statistical Analysis Plan (SAP) of the study. Primary and secondary efficacy variables will be analysed in the 'Full Analysis Set' according to the 'intention-to-treat' principle. Additionally, a per-protocol analysis will be also carried out in order to assess the robustness of the results. Cumulative incidences of the primary end point will be described using Kaplan-Meier estimates. A Cox regression model including site and other pre-defined baseline covariates, will be used to determine the hazard ratio for the primary end point and its 95% confidence interval. Further details will be provided in SAP of the study.

There will be two interim analyses and one final analysis, which will be undertaken on the intention-to-treat population. The interim evaluations will be performed by the independent Safety Committee on an annual basis (i.e. after the last randomized patient will complete one-year and two-years follow-up). The method of O'Brien and Fleming will be used to determine the threshold for statistical significance at the interim evaluation, that would constitute grounds to recommend trial termination. For the first year, this threshold will be a P value of 0.0006 (nominal significance level) or less; for the second year, it will be a P value of 0.015 (nominal significance level) or less; and for the third year (i.e. final analysis) it will be a P value of 0.047 or less, in order to 'preserve' an overall 5% level of significance. A further evaluation will take place at the end of the enrolment period for the purposes of possible adaptation of the sample size or observation period in order to achieve adequate power. During the interim analyses, in addition to the primary efficacy variable (i.e. development of CAN) the following secondary safety variables will also be considered: incidence of acute rejections and of tubulitis at one year and incidence of biopsy-proven acute

rejections at two years. These secondary comparisons will be done according to a two-sided test: however this comparison will be done for safety reasons and therefore there will be no adjustment for multiple testing.
Assessment of ‘equivalency’ of the two treatments and of emerging negative trends will be done considering a minimum important absolute difference equal to 10%. Other clinical criteria for stopping the study are provided in the Paragraph ‘Study Design’. Other details regarding the statistical approaches will be supplemented by a detailed plan which will be prepared after release of the protocol but before the start of the clinical part of the study. Should new evidence come to light as a result of running other trials before this trial is analyzed or should new methods of analysis become available in the statistical literature, or should the trial be affected by unforeseen circumstances, it may be necessary to modify the analysis plan.

STUDY ORGANIZATION

The study will be coordinated by the Clinical Research Center for Rare Diseases of the Mario Negri Institute for Pharmacological Research, Ranica, Bergamo and will be conducted with the cooperation of the Transplant Centers of Bergamo, Florence, Milan, Padua, Palermo, Varese, and the other Italian Transplant Centers included in the MYSS trial[17]. Data handling will be performed by Mario Negri Institute (MNI) Coordinating Center.
The study will be overseen by Steering and Safety Committees. The Steering Committee will be chaired by the principal investigator and will include three voting members (the chair and two external nephrologists) and four non-voting members (one statistician and three medical doctors). The Safety Committee will include three independent external reviewers (two nephrologists and one statistician). The Laboratory of Biostatistics of the Mario Negri Institute will prepare the randomization code that will be transmitted to an external unblinded data center that will periodically report the major clinical and adverse events to the safety committee.

DATA HANDLING AND RECORD KEEPING

Case report forms completion

CRFs will supplied by MNI Coordinating Center. Demographic, efficacy and safety data will be collected for the purpose of the study, to be documented by the investigator or his/her designated on the individual case report form (CRF). This also applies to the data for patients who, after having consented to participate, underwent baseline examinations but were not further randomized. The

investigator must be provided with a CRF copy. To ensure CRF legibility, CRFs should be filled out in block capitals with a black/blue ball point pen. Any CRF corrections must be carried out by the investigator. The correction has to be dated and initialled. A reasonable explanation must be given by the investigator for all missing data. All medical records upon which the CRFs are based must be kept for at least 15 years after completion of the study.. The investigator will be responsible for the accuracy of the data entered in the CRFs. All entries must be written in English in black ink. Source documents should be available to support all the data recorded in the CRFs.
Location of source data, including those for which the CRF might be accepted as being the sole source document, will be specified and listed at the Center initiation visit.
The CRF must be available for review/collection to designated MNI Coordinating Center Representatives at each scheduled monitoring visit.

Study monitoring

The study will be monitored by the staff of the Mario Negri Institute Drug Monitoring Unit. The Investigator agrees to allow monitoring visits on site (clinical area and laboratory) at regular times prior, during and after the completion of the study. These visits are designed to implement Good Clinical Practice requirements.
The study will be monitored by means of on-site visits, telephone calls and regular inspection of the case report forms with sufficient frequency to inspect the following: patient enrolment, compliance with the protocol procedures, the completeness and exactness of data entered on the case report forms, verification against original source documents, and occurrence of adverse events. Data verification is required and will be done by direct comparison with source documents in case of patient's respective consent or by cross-checking with source documents always giving due consideration to data protection and medical confidentiality.
The Investigator will ensure that adequate time will be available for the Clinical Monitor to review case report forms, study source and raw data, and to assure the accuracy and completeness of the recorded data. The Clinical Monitor will compare the data in the case report forms with source document(s).
During the visits, the Clinical Monitor will ensure that there is adherence to the protocol, and that patients included in the study meet the inclusion criteria and that patients and/or caregivers have given their written informed consent. The Clinical Monitor will collect completed and signed case report forms as they become available.

Data Management

In order to ensure that the data base accurately reflects data of the case report forms, a double entry procedure will be used.

The data entry will be made by the staff of the Mario Negri Institute Drug Monitoring Unit. Data will be entered by different staff members in the same data base. Inconsistencies derived from a computer-assisted comparison of these two data entries will be carefully clarified. In addition, a systematic and extensive electronic validation of the quality of the data base will take place.

Pre- and concomitant medication, concurrent diseases as well as adverse events will be coded according to standardized dictionaries. Once all data discrepancies will be corrected, the database will be locked and data converted into SAS-transport files.

Essential document retention

The investigator will retain copies of all the essential documents as required by the applicable regulatory requirements. The investigator should take measures to prevent accidental or premature destruction of these documents.

The essential documents include at least: the signed protocol, copies of the completed CRFs, signed patient informed consent documents from all patients who consented, Hospital records, and other source documents, IEC approval and all related correspondence, including approved documents, and all other documentation included in the investigator site file and pharmacy/dispensing file.

ADVERSE EVENT DEFINITION

Any untoward medical occurrence in a patient or clinical investigation subject administered a pharmaceutical product and which does not necessarily have to have a causal relationship with this treatment. An adverse event (AE) can therefore be any unfavourable and unintended sign (including an abnormal laboratory finding, for example), symptom, or disease temporally associated with the use of a medicinal product, whether or not considered related to the medicinal product..

Adverse Drug Reaction

An adverse drug reaction (ADR) is a noxious and unintended response to a medicinal product related to any doses. The phrase “response to a medicinal product” means that a causal relationship between a medicinal product and an adverse event is at least a reasonable possibility, i.e. the relationship cannot be ruled out.

Serious Adverse Event

A serious adverse event is any untoward medical occurrence that at any dose:

- results in death,
- is immediately life-threatening,

  NOTE: the term "life-threatening" in the definition of "serious" refers to an event in which the patient was at risk of death at the time of the event; it does not refer to an event which hypothetically might have caused death if it were more severe.
- requires inpatient hospitalisation or prolongation of existing hospitalisation,

  a) elective surgery or cases where the decision to hospitalize the patient was made before the signed consent or attendance at hospital without an overnight stay, is not hospitalisation

  b) event which would have normally required hospitalisation, but the patient was not hospitalised (for whatever reason) is hospitalisation

  c) if the patient was already in the hospital when the event occurred, and the event required inpatient treatment (i.e. the patient would have been hospitalized unless she/he already was) but did not cause the prolongation of the hospitalization, the event must be reported as serious for hospitalization;
- results in persistent or significant disability/incapacity, or
- is a congenital malformation/birth defect.
- other: it is a category that covers events that are considered as serious, when based upon appropriate medical judgment that they may jeopardise the patient/subject, and may require medical or surgical intervention to prevent one of the other seriousness criteria from occurring;
  - patient requiring treatment in order to prevent serious outcome: if a patient required treatment but not hospitalisation in order to prevent permanent and/or severe disablement or fatal outcome, then the event is reported per serious standard. Treatment may involve a physician's visit or presentation to the emergency room.
  - cancer: if cancer is the indication for treatment , only cancers of new histology and cases where there is clear evidence of exacerbation of an existing cancer qualify as a serious event. Every new occurrence of cancer must be reported as a serious event regardless of the duration between discontinuation of the drug and the occurrence of the cancer.
  - abuse or dependency: it qualifies as serious for other medical reasons

An event which doesn't meet these definitions is considered to be "Not Serious".

Collection/Registration of information

Adverse events will be monitored at each visit and by telephone reports from responsible caregivers as needed between outpatient visits.

Any adverse event reported spontaneously or upon questioning will be evaluated by the Investigator and recorded on the relevant page in the case report form.

However, if the event includes a group of signs and symptoms, which, when considered together, are known or presumed to characterize a syndrome, only one form must be filled in. In this case, all events will be fully described, but any additional information such as intensity, outcome, causality, etc., will refer to the syndrome only. For each adverse event the Investigator is required to evaluate the nature of the event, the date and time of its onset (if known), the date and time the adverse event stopped (if known), the duration of the event and its maximum intensity. The Investigator will record any remedial action taken and the final outcome of the adverse event. All modern facilities for resuscitation must be immediately available in case of emergency. The Investigator is also required to assess the causal relationship between the event and the study treatment.

All the patients experiencing adverse events must be monitored until symptoms and any abnormal laboratory values have returned to baseline, or until there is a satisfactory explanation for the changes observed, or until death, in which case a full pathologist's report should be supplied, if possible. All findings must be recorded on the "adverse event" page in the case report forms and in the patients' medical records.

In case of serious adverse events, the following reporting procedure has to be followed.

For all serious adverse events which occur during the study whether considered to be associated with the trial drug or not, the Investigator must complete the "Serious Adverse Event Report in Clinical Trial" and its "Addendum".

The initial SAE report must immediately be sent (within 24 hours from the knowledge of the event), by fax to Mario Negri Institute:.

The contact addresses for reporting of serious adverse events is:

Nadia Rubis, *Clinical Monitor*

Drug Monitoring Unit

Istituto di Ricerche Farmacologiche Mario Negri

Centro di Ricerche Cliniche per le Malattie Rare

Aldo e Cele Daccò

Via G. B. Camozzi, 3

I-24020 Ranica (Bergamo)

Italy

SAEs Fax: +39 035 4535371

Tel: +39 035 45351

e-mail: farmacovigilanza.vc@marionegri.it

LEGAL AND ETHICAL ASPECTS

Health Authorities and Ethics Committee

The study protocol will be in accordance with the declaration of Helsinki and will be approved by the institutional review board at each center and by the safety committee of the study group. The study protocol and all the other appropriate documents will be submitted to the Regulatory Authorities in accordance with local legal requirements. The investigational sites will not commence study procedures until has received the appropriate written approval. All protocol amendments, treatment-related serious adverse events and new versions of the Investigator's Brochure must be submitted to the Principal Investigators and to the Ethics Committee.

Written Informed Consent

Good Clinical Practice guidelines require that a written informed consent be obtained from each patient. Doctor must inform patient and caregiver of the aims of the study and how it will be organized, the type of study treatments, the anticipated benefits which can be expected from the study, any potential hazards of the study and discomfort it may entail, alternative treatments, the freedom to ask for further information at any time, the patient's right to withdraw from the trial at any time without giving reasons and without jeopardizing the further course of the treatment, the existence of patient's insurance cover and obligations following from this cover. This information should be given in both oral and written form. The patient and the caregiver should have sufficient time to decide whether or not to take part in the study.

The study drugs are currently used in clinical practice to prevent acute rejection in kidney transplant recipients. The main risk of this immunosuppressive protocol is the occurrence of acute rejection. This risk will be minimized by excluding patients at high immunological risk, such as recipients of previous transplants or those with a PRA > 10%. Graft function will be closely monitored not only at planned visits, but also after any change in immunosuppressive therapy and up to complete resolution of any clinical or laboratory abnormality, and whenever deemed appropriate by the investigators. The steering and ethical committees of the study will be periodically updated about the incidence of major adverse events including, in particular, acute rejection episodes that may occur during or after CsA withdrawal. Thus, the ethical committee will have the possibility to monitor the incidence of acute rejections and their outcomes (such as response to steroid therapy

and renal function recovery) and prematurely stop the study whenever deemed appropriate due to an excessive incidence of events or of rejection-related sequelae).

Insurance policy

The study is covered by an insurance policy, according to the laws in force, in the event of a patient suffering any significant deterioration in health or well-being, which is proven as being as a direct result of study participation.

REFERENCES

1. McDevitt LM. Immunosuppressive agents on the horizon. J Pharm Practice 2003;16:434.
2. Olyaei AJ, de Mattos AM, Bennett WM. Nephrotoxicity of immunosuppressive drugs: new insight and preventive strategies. Curr Opin Crit Care. 2001;7(6):384.
3. Land W, Vincenti F. Toxicity-sparing protocols using mycophenolate mofetil in renal transplantation. Transplantation 2005;80:S221.
4. Keogh A. Calcineurin inhibitors in heart transplantation. J Heart Lung Transplant 2004;23:S202
5. Hocker B, John U, Plank C et al. Successful withdrawal of steroids in pediatric renal transplant recipients receiving cyclosporine A and mycophenolate mofetil treatment: results after four years. Transplantation 2004;78:228.
6. Nankivell BJ; Borrows RJ, Fung CL, et al. Calcineurin inhibitor nephrotoxicity: longitudinal assessment by protocol histology. Transplantation 2004;78:557.
7. Knechtle SJ, Vargo D, Fechner J, et al. FN18-CRM9 immunotoxin promotes tolerance in primate renal allografts. Transplantation 1997;63:1.
8. Thomas JM, Neville DM, Contreras JL, et al. Preclinical studies of allograft tolerance in rhesus monkeys: a novel anti-CD3-immunotoxin given peritransplant with donor bone marrow induces operational tolerance to kidney allografts. Transplantation 1997;64:124.
9. Ruggenenti P, Codreanu I, Cravedi P et al. Basiliximab combined to low-dose RATG: a possibile furthe step toward effective and minimally toxic T cell targeted therapy in kidney transplantation. Clin J Am Soc Nephrol 2006;1:546.
10. Olyaei AJ, de Mattos AM, Bennett WM. Nephrotoxicity of immunosuppressive drugs: new insight and preventive strategies. Curr Opin Crit Care. 2001;7(6):384.
11. Abramowicz D, Del Carmen Rial M, Vitko S, Del Castillo D, Manas D, Lao M, Gafner N, Wijngaard P. Cyclosporine withdrawal from a mycophenolate mofetil-containing immunosuppressive regimen: results of a five-year, prospective, randomized study. J Am Soc Nephrol. 2005;16(7):2234.

12. Dudley C, Pohanka E, Riad H, Dedochova J, Wijngaard P, Sutter C, Silva HT Jr; Mycophenolate Mofetil Creeping Creatinine Study Group. Mycophenolate mofetil substitution for cyclosporine a in renal transplant recipients with chronic progressive allograft dysfunction: the "creeping creatinine" study. Transplantation. 2005;79(4):466.
13. Afzali B, Shah S, Chowdhury P, O'sullivan H, Taylor J, Goldsmith D. Low-dose mycophenolate mofetil is an effective and safe treatment to permit phased reduction in calcineurin inhibitors in chronic allograft nephropathy. Transplantation. 2005;79(3):304.
14. Merville P, Berge F, Deminiere C, Morel D, Chong G, Durand D, Rostaing L, Mourad G, Potaux L. Lower incidence of chronic allograft nephropathy at 1 year post-transplantation in patients treated with mycophenolate mofetil. Am J Transplant. 2004;4(11):1769.
15. Ojo AO, Meier-Kriesche HU, Hanson JA: Mycophenolate mofetil reduces late renal allograft loss independent of acute rejection. Transplantation 2000;69(11):2405.
16. Chu SH, Wu CT, Chou CC, Chen Y, Liu KL, Chiang YJ. Clinical experience of mycophenolate mofetil in the treatment of chronic allograft nephropathy in kidney transplantation: three-year follow-up. Transplant Proc. 2004 ;36(7):2073.
17. Remuzzi G, Lesti M, Gotti E et al. Mycophenolate mofetil versus azathioprine for prevention of acute rejection in renal transplantation (MYSS): a randomised trial. Lancet 2004;364:503.
18. Keunecke C, Rothenpieler U, Zanker B, et al. Mycophenolate mofetil monotherapy: an example of a safe nephrotoxicity/atherogenicity-free immunosuppressive maintenance regimen in a selected group of kidney-transplanted patients. Transplant Proc 2000;32:S6
19. Vanrenterghem Y, Lebraunchu Y, Hené R, et al. Double-blind comparison of two corticosteroid regimens plus mycophenolate mofetil and cyclosporine for prevention of acute renal allograft rejection. Transplantation 2000;70:1352.
20. Racusen LC, Solez K, Colvin RB et al. The Banff 97 working classification of renal allograft pathology. Kidney intern 1999; 55: 713-723.
21. Remuzzi G, Cravedi P, Perna A et al. Long-term outcome of renal transplantation from older donors. New Engl J Med 2006; 354: 343-352.
22. The Tricontinental Mycophenolate Mofetil Renal Transplantation Study Group. A blinded, randomized clinical trial of mycophenolate mofetil for the prevention of acute rejection in cadaveric renal transplantation. Transplantation 1996; 61: 1029-1037.
23. Sollinger HW for the US Renal Transplant Mycophenolate Mofetil Study Group. Mycophenolate for the prevention of acute rejection in primary cadaveric renal allograft recipients. Transplantation 1995; 60: 225-232

Appendix 1

	PHASE A																														
Visit number	1	2	3	4	5	6	7	8	9	10	11	12	13	14	15	16	17	18	19	20	21	22	23	24	25	26	27	28	29	30	31
Time[1]	0 d	1 d	2 d	3 d	4 d	5 d	6 d	7 d	2 w	3 w	4 w	5 w	6 w	7 w	8 w	9 w	10 w	11 w	12 w	14 w	16 w	18 w	20 w	22 w	24 w	7 m	8 m	9 m	10 m	11 m	12 m
Baseline Data	X																														
Chest X-Ray	X																														
ECG at Rest	X																														x
Inclusion/Exclusion Criteria	X																														
Randomization																															
Complete Physical Examination	X							X	X	X	x	x	x	x	x	x	x	x	x	x	x	x	x	x	x	x	x	x	x	x	x
Blood Pressure-Pulse Rate-Weight	X	X	x	x	x	x	x	X	X	X	x	x	x	x	x	x	x	x	x	x	x	x	x	x	x	x	x	x	x	x	x
Blood Laboratory Examinations-1[2]	X																		x						x			x			x
Blood Laboratory Examinations-2[3]								X			x				x						x		x			x	x		x	x	
Blood Laboratory Examinations-3[4]		X	x	x	x	x	x		X	X		x	x	x		x	x	x		x		x		x							
Morning Urine Sample[5]									X	X	x	x	x	x	x	x	x	x	x	x	x	x	x	x	x	x	x	x	x	x	x
24-Hours Urine Collection[6]									X	X	x	x	x	x	x	x	x	x	x		x		x		x	x	x	x	x	x	x
Urine Culture								X	X	X	x	x	x	x	x	x	x	x	x	x	x	x	x	x	x	x	x	x	x	x	x
Graft Ultrasonography								X											x												x
Graft Biopsy	X																														x
CsA Level (C_0)		X	x	X	x	x	x	X	X	X	x		x		x		x		x		x		x		x	x	x	x	x	x	x
CsA Tapering																															x
MMF Level									X										x						x						X
Pregnancy Test	X																														
CMV Sierology	X										x	x	x	x	x	x	x	x	x	x											
HCV, HBsAg, HIV Sierology	X																														
Lymphocytes subpopulations	X							X			x				x				x						x			x			x
Study Drug Temporary Withdrawals		X	x	x	x	x	x	X	X	X	x	x	x	x	x	x	x	x	x	x	x	x	x	x	x	x	x	x	x	x	x
Study Drug Permanently Discontinuation		(x)	(x)	(x)	(x)	(x)	(x)	(x)	(x)	(x)	(x)	(x)	(x)	(x)	(x)	(x)	(x)	(x)	(x)	(x)	(x)	(x)	(x)	(x)	(x)	(x)	(x)	(x)	(x)	(x)	(x)
Concomitant Medication/Non-Drug Therapy	X	X	x	x	x	x	x	X	X	X	x	x	x	x	x	x	x	x	x	x	x	x	x	x	x	x	x	x	x	x	x
Adverse Events		X	x	x	x	x	x	X	X	X	x	x	x	x	x	x	x	x	x	x	x	x	x	x	x	x	x	x	x	x	x
Extra Visits		X	x	x	x	x	x	X	X	X	x	x	x	x	x	x	x	x	x	x	x	x	x	x	x	x	x	x	x	x	x
Trial End Summary		(x)	(x)	(x)	(x)	(x)	(x)	(x)	(x)	(x)	(x)	(x)	(x)	(x)	(x)	(x)	(x)	(x)	(x)	(x)	(x)	(x)	(x)	(x)	(x)	(x)	(x)	(x)	(x)	(x)	(x)

1 Time: "d" means days, "w" means week(s), "m" means months.

2 Blood laboratory examinations-1: creatinine, urea, sodium, potassium, glucose, HbA1c, ALT/GOT, AST/GPT, alkaline phosphatase, γGT, calcium, phosphate, uric acid, total cholesterol, LDL-cholesterol, HDL-cholesterol, triglycerides, venous pH, erithrocytes, hematocrit, hemoglobin, leukocytes with differential count, platelets HLA-antibodies (only at 12 month).

3 Blood laboratory examinations-2: creatinine, urea, sodium, potassium, glucose, ALT/GOT, AST/GPT, alkaline phosphatase, γGT, calcium, phosphate, uric acid, total cholesterol, LDL-cholesterol, HDL-cholesterol, triglycerides, venous pH, erithrocytes, hematocrit, hemoglobin, leukocytes with differential count, platelets.

4 Blood laboratory examinations-3: creatinine, urea, sodium, potassium, glucose, ALT/GOT, AST/GPT, alkaline phosphatase, γGT, venous pH, erithrocytes, hematocrit, hemoglobin, leukocytes with differential count, platelets.

5 Morning urine sample: Multistix 10SG.

6 24-hours urine excretions of albumin, total proteins, and 24-hours creatinine clearance.

Appendix 2

	Phase B																							
**Visit number**	32	33	34	35	36	37	38	39	40	41	42	43	44	45	46	47	48	49	50	51	52	53	54	55
Time (months)	13	14	15	16	17	18	19	20	21	22	23	24	25	26	27	28	29	30	31	32	33	34	35	36
Complete Physical Examination	x	x	x	x	x	x	x	x	x	x	x	x	x	x	x	x	x	x	x	x	x	x	x	x
Blood Pressure-Pulse Rate-Weight	x	x	x	x	x	x	x	x	x	x	x	x	x	x	x	x	x	x	x	x	x	x	x	x
Blood Laboratory Examinations-1[1]			x			x			x			x			x			x			x			x
Blood Laboratory Examinations-2[2]	x	x		x	x		x	x		x	x		x	x		x	x		x	x		x	x	
ECG at Rest												x												x
Morning Urine Sample[3]	x	x	x	x	x	x	x	x	x	x	x	x	x	x	x	x	x	x	x	x	x	x	x	x
3 Consecutive 24-Hours Urine Collections[4]	x	x	x	x	x	x	x	x	x	x	x	x	x	x	x	x	x	x	x	x	x	x	x	x
Urine Culture	x	x	x	x	x	x	x	x	x	x	x	x	x	x	x	x	x	x	x	x	x	x	x	x
Graft Ultrasonography												x												x
CSA Level (C_0)	x	x	x	x	x	x	X	X	x	x	x	x	x	x	x	x	x	x	(x)					
CSA Tapering	x	x	x	x	x	x							x	x	x	x	x	x						
CSA Permanently Withdrawal																		X						
Graft Biopsy												x												x
Lymphocytes subpopulations												x												x
Study Drug Temporary Withdrawals		x	x	x	x	x	x	x	x	x	x	x	x	x	x	x	x	x	x	x	x	x	x	x
Study Drug Permanently Discontinuation		(x)	(x)	(x)	(x)	(x)	(x)	(x)	(x)	(x)	(x)	(x)	(x)	(x)	(x)	(x)	(x)	(x)	(x)	(x)	(x)	(x)	(x)	(x)
Concomitant Medication/Non-Drug Therapy	x	x	x	x	x	x	x	x	x	x	x	x	x	x	x	x	x	x	x	x	x	x	x	x
Adverse Events	x	x	x	x	x	x	x	x	x	x	x	x	x	x	x	x	x	x	x	x	x	x	x	x
Extra Visits	x	x	x	x	x	x	x	x	x	x	x	x	x	x	x	x	x	x	x	x	x	x	x	x
Trial End Summary	(x)	(x)	(x)	(x)	(x)	(x)	(x)	(x)	(x)	(x)	(x)	(x)	(x)	(x)	(x)	(x)	(x)	(x)	(x)	(x)	(x)	(x)	(x)	x

1 **Blood laboratory examinations-1:** creatinine, urea, sodium, potassium, glucose, HbA1c, ALT/GOT, AST/GPT, alkaline phosphatase, γGT, calcium, phosphate, uric acid, total cholesterol, LDL-cholesterol, HDL-cholesterol, triglycerides, venous pH, erythrocytes, hematocrit, haemoglobin, leukocytes with differential count, platelets, HLA-antibodies (only at 18, 24 and 30 month).

2 **Blood laboratory examinations-2:** creatinine, urea, sodium, potassium, glucose, ALT/GOT, AST/GPT, alkaline phosphatase, γGT, calcium, phosphate, uric acid, total cholesterol, LDL-cholesterol, HDL-cholesterol, triglycerides, venous pH, erythrocytes, hematocrit, haemoglobin, leukocytes with differential count, platelets.

3 **Morning urine sample:** Multistix 10SG.

4 **3 consecutive 24-hours urine collections:** excretions of albumin, creatinine, potassium, total proteins, sodium, urea; creatinine clearance (3rd collection only)

Appendix 3

	Follow-Up			
Visit number	56	57	58	59
Time (months)	39	42	45	48
Complete Physical Examination	x	x	x	x
Blood Pressure-Pulse Rate-Weight	x	x	x	x
Blood Laboratory Examinations-1[1]	x	x	x	x
ECG at Rest				x
Morning Urine Sample[2]	x	x	x	x
3 Consecutive 24-Hours Urine Collections[3]	x	x	x	x
Urine Culture	x	x	x	x
Graft Ultrasonography				x
Study Drug Temporary Withdrawals	x	x	x	x
Study Drug Permanently Discontinuation	(x)	(x)	(x)	(x)
Concomitant Medication/Non-Drug Therapy	x	x	x	x
Adverse Events	x	x	x	x
Extra Visits	x	x	x	x
Trial End Summary	(x)	(x)	(x)	(x)

1 Blood laboratory examinations-1: creatinine, urea, sodium, potassium, glucose, HbA1c, ALT/GOT, AST/GPT, alkaline phosphatase, γGT, calcium, phosphate, uric acid, total cholesterol, LDL-cholesterol, HDL-cholesterol, triglycerides, venous pH, erithrocytes, hematocrit, hemoglobin, leukocytes with differential count, platelets.

2 Morning urine sample: Multistix 10SG.

3 3 consecutive 24-hours urine collections: excretions of albumin, creatinine, potassium, total proteins, sodium, urea; creatinine clearance (3rd collection only).

Appendix

MONITORING OF ACUTE REJECTION EPISODES AND STOPPING RULES
DURING THE CYCLOSPORIN TAPERING AND WITHDRAWAL PHASE

Monitoring and treatment
Serum creatinine will be measured every week during cyclosporin tapering, for at least two weeks after cyclosporin withdrawal and every month thereafter (while on stable monotherapy). Additional measurements will be performed whenever deemed appropriate on clinical grounds. Should serum creatinine increase by 0.3 mg/dl or more between two consecutive measurements, the test will be repeated. If the increase is confirmed, and a kidney ultrasound evaluation excludes urinary tract obstruction or vascular complications, a kidney biopsy will be performed. Biopsy-proven rejections will be treated according to centre's practice.

Reporting, analysis and stopping rules
Each acute rejection episode, as well as any other serious adverse event, will be reported to the coordinating centre within 24 hours. Every month the cordinating center will report all incident rejection episodes to an Independent Safety Panel that will include two nephrologists and one statistician not directly involved in the trial. The Safety Panel will review the reported events and will have the authority to stop the trial if the cumulative incidence of acute rejections during cyclosporin tapering and withdrawal will exceed 15% (a) or for any other relevant safety reason. Should data show a difference in acute rejections between the two treatment arms exceeding 30%, the Panel will also have the authority to reallocate all patients to the most effective treatment, without stopping the trial.

a. Remuzzi G et al. Mycophenolate mofetil versus azathioprine for prevention of acute rejection in renal transplantation (MYSS): a randomised trial. Lancet 2004,364: 503-512
