# Supplementary material for: Mycophenolate mofetil versus azathioprine in kidney transplant recipients on steroid-free, low-dose cyclosporine immunosuppression (ATHENA): A pragmatic randomized trial
Source: PLoS Med. 2021 Jun 24;18(6):e1003668. doi: 10.1371/journal.pmed.1003668 (PMC8224852; doi:10.1371/journal.pmed.1003668)
Supplement: S3 Text — (DOC) [file pmed.1003668.s003.doc]

CONSORT 2010 checklist of information to include when reporting a randomised trial*

| Section/Topic | Item No | Checklist item | Reported on page No |
| --- | --- | --- | --- |
| Title and abstract | | | |
|  | 1a | Identification as a randomised trial in the title | 1  (title:  “A pragmatic randomized trial”) |
|  | 1b | Structured summary of trial design, methods, results, and conclusions (for specific guidance see CONSORT for abstracts) | 3-4  Abstract |
| Introduction, Paragraph 1 | | | |
| Background and objectives | 2a | Scientific background and explanation of rationale | 7-9  “Introduction” Section |
|  | 2b | Specific objectives or hypotheses | 7-9  “Introduction” Section  10  “Methods” Section  “Prospective Protocol and analysis plan”  Heading  14  “Outcome measures” Heading |
| Methods, Paragraph 2 | | | |
| Trial design | 3a | Description of trial design (such as parallel, factorial) including allocation ratio | 3  Abstract  10  “Methods” Section  “Prospective Protocol and analysis plan”  Heading |
|  | 3b | Important changes to methods after trial commencement (such as eligibility criteria), with reasons | S2 Appendix  Study Protocol and amendments |
| Participants | 4a | Eligibility criteria for participants | 3  Abstract  10-11  “Methods” Section  “Participants, setting and ethics”  Heading |
|  | 4b | Settings and locations where the data were collected | 3  Abstract  10-11  “Methods” Section  “Participants, setting and ethics”  Heading  S1 Appendix  “Athena Study Organization” |
| Interventions | 5 | The interventions for each group with sufficient details to allow replication, including how and when they were actually administered | 3  Abstract  10  “Methods” Section  “Prospective Protocol and analysis plan”  Heading  S2 Appendix  Study Protocol and amendments |
| Outcomes | 6a | Completely defined pre-specified primary and secondary outcome measures, including how and when they were assessed | 14  “Methods” Section  “Outcome measures” Heading  S2 Study Protocol and amendments |
|  | 6b | Any changes to trial outcomes after the trial commenced, with reasons | NA |
| Sample size | 7a | How sample size was determined | 14-15  “Methods” Section  “Sample size and statistical analyses” Heading  S2 Study Protocol and amendments |
|  | 7b | When applicable, explanation of any interim analyses and stopping guidelines | 15  “Methods” Section  “Sample size and statistical analyses” Heading  S2 Study Protocol and amendments |
| Randomisation: |  |  |  |
|  |  |  |  |
| Sequence generation | 8a | Method used to generate the random allocation sequence | 11-12  “Methods” Section  “Randomization” Heading |
|  | 8b | Type of randomisation; details of any restriction (such as blocking and block size) | 11-12  “Methods” Section  “Randomization” Heading |
| Allocation concealment mechanism | 9 | Mechanism used to implement the random allocation sequence (such as sequentially numbered containers), describing any steps taken to conceal the sequence until interventions were assigned | 11-12  “Methods” Section  “Randomization” Heading |
| Implementation | 10 | Who generated the random allocation sequence, who enrolled participants, and who assigned participants to interventions | 11  “Methods” Section  “Randomization” Heading |
| Blinding | 11a | If done, who was blinded after assignment to interventions (for example, participants, care providers, those assessing outcomes) and how | 11-12  “Methods” Section  “Randomization” Heading |
|  | 11b | If relevant, description of the similarity of interventions | NA |
| Statistical methods | 12a | Statistical methods used to compare groups for primary and secondary outcomes | 14-15  “Methods” Section  “Sample size and statistical analyses” Heading |
|  | 12b | Methods for additional analyses, such as subgroup analyses and adjusted analyses | 15  “Methods” Section  “Sample size and statistical analyses” Heading |
| Results, Paragraph 3 | | | |
| Participant flow (a diagram is strongly recommended) | 13a | For each group, the numbers of participants who were randomly assigned, received intended treatment, and were analysed for the primary outcome | 16-17  “Results” Section  “Patients” Heading  Fig. 1 (Participant flow-chart) |
|  | 13b | For each group, losses and exclusions after randomisation, together with reasons | 16-17  “Results” Section  “Patients” Heading  Fig. 1 (Participant flow-chart) |
| Recruitment | 14a | Dates defining the periods of recruitment and follow-up | 16  “Results” Section  “Patients” Heading |
|  | 14b | Why the trial ended or was stopped | NA |
| Baseline data | 15 | A table showing baseline demographic and clinical characteristics for each group | Tables 1, 2 |
| Numbers analysed | 16 | For each group, number of participants (denominator) included in each analysis and whether the analysis was by original assigned groups | 3  Abstract  16-27  “Results” Section  Fig 1-3  Tables S1, S2 |
| Outcomes and estimation | 17a | For each primary and secondary outcome, results for each group, and the estimated effect size and its precision (such as 95% confidence interval) | 3  Abstract  16-25  “Results” Section  Fig 2-3,  Table S2 |
|  | 17b | For binary outcomes, presentation of both absolute and relative effect sizes is recommended | 3  Abstract  16-25  “Results” Section  Table 5, S2 |
| Ancillary analyses | 18 | Results of any other analyses performed, including subgroup analyses and adjusted analyses, distinguishing pre-specified from exploratory | 25  “Results” Section  “Immunosuppressive therapy nad concomitant medications”  Table S1 |
| Harms | 19 | All important harms or unintended effects in each group (for specific guidance see CONSORT for harms) | 26-27  “Results” Section  Tables 4,5 |
| Discussion, Paragraph 4 | | | |
| Limitations | 20 | Trial limitations, addressing sources of potential bias, imprecision, and, if relevant, multiplicity of analyses | 31-32  “Discussion” Section  “Study limitations and mitigating factors” |
| Generalisability | 21 | Generalisability (external validity, applicability) of the trial findings | 31-32  “Discussion” Section  “Study limitations and mitigating factors” |
| Interpretation | 22 | Interpretation consistent with results, balancing benefits and harms, and considering other relevant evidence | 28-33  “Discussion” Section |
| Other information | | |  |
| Registration | 23 | Registration number and name of trial registry | 1  (“Registry and Identifier”) |
| Protocol | 24 | Where the full trial protocol can be accessed, if available | S2 Study Protocol and amendments |
| Funding | 25 | Sources of funding and other support (such as supply of drugs), role of funders | 34  “Aknowledgments” Section |

*We strongly recommend reading this statement in conjunction with the CONSORT 2010 Explanation and Elaboration for important clarifications on all the items. If relevant, we also recommend reading CONSORT extensions for cluster randomised trials, non-inferiority and equivalence trials, non-pharmacological treatments, herbal interventions, and pragmatic trials. Additional extensions are forthcoming: for those and for up to date references relevant to this checklist, see [www.consort-statement.org](http://www.consort-statement.org/).
