# Supplementary material for: Mycophenolate mofetil versus azathioprine in kidney transplant recipients on steroid-free, low-dose cyclosporine immunosuppression (ATHENA): A pragmatic randomized trial
Source: PLoS Med. 2021 Jun 24;18(6):e1003668. doi: 10.1371/journal.pmed.1003668 (PMC8224852; doi:10.1371/journal.pmed.1003668)
Supplement: S4 Text — (PDF) [file pmed.1003668.s004.pdf]

All.1/o

Comitato di Bioetica dell'Azienda Ospedali Riuniti di Bergamo
Largo Barozzi,1- 24128 Bergamo

Il Comitato di Bioetica riunito in data 13 novembre 2006per esprimere il proprio parere sul protocollo :

"A randomized, prospective, multicentre trial to compare the effect on chronic allograft nephropathy prevention of mycofenolate mofetil versus azathioprine as the sole immunosuppressive therapy for kidney transplant recipients" MAMMOUTH
EudraCT N. 2006-005604-14

con il medicinale/i : *Azatioprina e Micofenolato Mofetil*

Richiesta di autorizzazione presentata dal *dott. Giuseppe Remuzzi* direttore dell' *U.S.C. di Nefrologia e Dialisi*

Promotore: *; Istituto di Ricerche Farmacologiche "M. Negri"- finanziato da AIFA (proposta 2.21 cod. FARM5PWB9L)*

VERIFICATO

il numero legale (come dal elenco allegato) e che i Componenti del C.d.B per i quali sussiste un conflitto di interesse di tipo diretto e indiretto si astengano nel pronunciare un parere

VALUTATI I SEGUENTI DOCUMENTI :

1. Protocollo versione june 12th 2006
2. Sintesi della proposta di sperimentazione + elenco dei centri partecipanti
3. Foglio Informativo / consenso informato versione 09/10/2006
4. Informativa al medico di medicina generale versione 09/10/2006
5. Dichiarazione di assunzione di responsabilità
6. Dichiarazione di comunicazione degli eventi avversi seri o inattesi
7. Dichiarzione pubblica sul conflitto di interessi (All. 2 del decreto 17/12/04)

TIPOLOGIA DELLO STUDIO, OBIETTIVI:
studio di fase IV, in aperto, randomizzato, multicentrico e nazionale il cui obiettivo è confrontare l'effetto di micofenolato mofetil e azatioprina impiegati in monoterapia immunosoppressiva cronica nello sviluppo di rigetto cronico in trapiantati di rene che ricevono un induzione con basiliximab a basse dosi di RATG (Rabbit Anti-human Thyocyte Globulin).
Localmente verranno arruolati 40 malati (8 centri per un totale di 224 malati)
Documentazione trasmessa al C.d.B in data 23/10/2006

RISULTATI DELLA VALUTAZIONE:
Il Comitato di Bioetica, esaminata la documentazione indicata sopra,

HA ESPRESSO, AI SENSI DEL DECRETO LEGISLATIVO N. 211/2003,
PARERE UNICO FAVOREVOLE ALL'EFFETTUAZIONE DELLA SPERIMENTAZIONE CLINICA

In osservanza a quanto previsto dal Decreto Legislativo n.211 del 24/08/2003 *"Attuazione della direttiva 2001/20/CE relativa all'applicazione della buona pratica clinica nell'esecuzione delle sperimentazioni cliniche di medicinali per uso clinico"*, dal D.M 15.07.1997 *"Recepimento delle linee guida dell'Unione Europea di buona pratica clinica per l'esecuzione delle sperimentazioni cliniche dei medicinali"* e dai D.M 18.03.1998 *"Linee guida di riferimento per l'istituzione ed il funzionamento dei Comitati Etici" e Modalità per l'esenzione dagli accertamenti sui medicinali utilizzati nelle sperimentazioni cliniche"*.
Il parere è stato espresso a maggioranza semplice dei membri presenti alla seduta del Comitato Etico.
Si richiede che questo C.d.B venga informato dell'inizio della sperimentazione e della sua conclusione o eventuale interruzione.
Il Responsabile della sperimentazione dovrà far pervenire, inoltre, una relazione annuale sullo stato della sperimentazione.
Inoltre, il C.d.B dovrà essere informato, unitamente al Ministero della Salute di ogni successivo emendamento al protocollo e degli eventi avversi, seri o inattesi, insorti nel corso dello studio che potrebbero influire sulla sicurezza dei soggetti o sul proseguimento dello studio. Si ribadisce che i costi della sperimentazione non dovranno gravare sul SSN come previsto al comma 5.1 dell'allegato 1 del D.M 18/03/98 e art.20 del D. Legislativo n.211 del 24 giugno 2003.

Il Segretario del C.d.B
(Dott. E. Locati)

Direzione Sanitaria **Comitato di Bioetica**

FOGLIO PRESENZE

Bergamo, 13 novembre 2006

Presidente Comitato Etico<br>Prof. Sandro Spinsanti<br>(Direttore Istituto Giano – Roma)	[signature]
Dott. Marco Salmoiraghi<br>(Direttore Sanitario Aziendale)	ASSENTE
Dott.ssa Cristina Agostinis<br>A.O. Ospedali Riuniti di Bergamo<br>(Dirigente Medico USC Neuroradiologia)	[signature]
Avv. Mauro Angarano<br>(Esperto in materie giuridiche)	ASSENTE
Dott. Tullio Bertani<br>A.O. Ospedali Riuniti di Bergamo<br>(Dirigente Medico USC Nefrologia e Dialisi)	[signature]
Dott. Virginio Bonito<br>A.O. Ospedali Riuniti di Bergamo<br>(Dirigente Medico USC Neurologia)	[signature]
Dott. Daniele Cafini<br>A.O. Ospedali Riuniti di Bergamo<br>(Direttore USC Medicina Legale)	ASSENTE
Sig.ra M. Teresa Carlessi<br>A.O. Ospedali Riuniti di Bergamo<br>(Capo Sala Referente Area Medicina Generale)	ASSENTE
Don Maurizio Chiodi<br>Docente di Teologia Morale Facoltà Teologica<br>Italia Settentrionale Milano-Bergamo	[signature]
Sig.ra Lina Colleoni<br>A.O. Ospedali Riuniti di Bergamo<br>(Capo Sala Direzione Infermieristica e Tecnico-sanitaria, Riabilitativa e Ostetrica)	Lina Colleoni
Dott. Claudio Farina<br>A.O. Ospedale S. Carlo Milano<br>(Direttore USC Microbiologia)	[signature]

Dott. Albino Fascendini
(Esperto in Bioetica)

Dott. Tiziano Gamba
(Medico di Medicina Generale Territoriale)

Dott. Roberto Labianca
A.O. Ospedali Riuniti di Bergamo
(Direttore USC Oncologia Medica) ASSENTE

Dott. Francesco Locati
A.O. Ospedali Riuniti di Bergamo
(Dirigente Medico Direzione Sanitaria)

Dr.ssa Monia Lorini
A.O. Ospedali Riuniti di Bergamo
(Dirigente Farmacista USC Farmacia)

Dott. Franco Maggiolo
A.O. Ospedali Riuniti di Bergamo
(Biostatistico, Dirigente Medico USC Malattie Infettive) ASSENTE

Dott. Gianmariano Marchesi
A.O. Bolognini di Seriate
(Direttore USC Anestesia e Rianimazione) ASSENTE

Dott. Giulio Mingardi
A.O. Ospedali Riuniti di Bergamo
(Dirigente Medico USC Nefrologia e Dialisi)

Dr.ssa Marcella Montini
A.O. Ospedali Riuniti di Bergamo
(Dirigente Medico USC Endocrinologia)

Ost. Edda Pellegrini
A.O. Ospedali Riuniti di Bergamo
(Ostetricia, Coordinatore Corso di Laurea in Ostetricia)

Cav. Leonida Pozzi
(Rappresentante Associazioni di Volontariato)

Dott. Arrigo Schieppati
A.O. Ospedali Riuniti di Bergamo
(Dirigente Medico USC Nefrologia e Dialisi)

Dr.ssa M. Simonetta Spada
A.O. Ospedali Riuniti di Bergamo
(Dirigente Psicologo USD Psicologia Clinica)

Dr. G. Carlo Taddei
A.O. Ospedali Riuniti di Bergamo
(Direttore USC Farmacia) ASSENTE

Dott.ssa Anna Maria Tempra
A.O. Ospedali Riuniti di Bergamo
(Dirigente Medico USC Patologia Neonatale)

Dr.ssa Leonarda Torre
A.O. Ospedali Riuniti di Bergamo
(Dirigente Biologo Direzione Sanitaria)
