# Supplementary material for: Mycophenolate mofetil versus azathioprine in kidney transplant recipients on steroid-free, low-dose cyclosporine immunosuppression (ATHENA): A pragmatic randomized trial
Source: PLoS Med. 2021 Jun 24;18(6):e1003668. doi: 10.1371/journal.pmed.1003668 (PMC8224852; doi:10.1371/journal.pmed.1003668)
Supplement: S1 Table — (DOCX) [file pmed.1003668.s005.docx]

**S1 Table 1.** Concomitant medications throughout the study period.

|  | AZA  *(n=114)* | MMF  *(n=119)* |
| --- | --- | --- |
| ACE inhibitors alone | 46 (40%) | 46 (39%) |
| ACE inhibitors in combination with other antihypertensive agents | 3 (2.6%) | 2 (1.7%) |
| ARB alone | 16 (14%) | 16 (13%) |
| ARB in combination with other antihypertensive agents | 2 (1.7%) | 2 (1.7%) |
|  |  |  |
| Statins | 58 (51%) | 47 (39%) |
| Other lipid-lowering agents | 50 (44%) | 42 (35%) |
|  |  |  |
| Antithrombotic agents | 91 (80%) | 85 (71%) |
| Platelet aggregation inhibitors | 60 (53%) | 45 (38%)* |
| Heparin | 65 (57%) | 57 (48%) |
| Other antithrombotic agents | 12 (11%) | 7 (5.9%) |

Chi Squared or Fisher's Exact Test: * p<0.05. ACE, angiotensin-converting enzyme. ARB, angiotensin receptor blockers.
