# Supplementary material for: Mycophenolate mofetil versus azathioprine in kidney transplant recipients on steroid-free, low-dose cyclosporine immunosuppression (ATHENA): A pragmatic randomized trial
Source: PLoS Med. 2021 Jun 24;18(6):e1003668. doi: 10.1371/journal.pmed.1003668 (PMC8224852; doi:10.1371/journal.pmed.1003668)
Supplement: S2 Table — (DOCX) [file pmed.1003668.s006.docx]

**S2 Table 2**. Acute rejections in the study group considered as a whole (Overall) and in the two treatment groups.

|  | Overall  *(n=233)* | AZA  *(n=114)* | MMF  *(n=119)* | HR  95%CI | P value |
| --- | --- | --- | --- | --- | --- |
| Biopsy-proven | 54 (23.2) | 34 (29.8) | 20( 16.8) | 0.58 (0.34 to 1.02) | *0.057* |
| Sub-clinical | 35 (15.0) | 14 (12.3) | 21 (17.6) | 1.49 (0.76 to 2.92) | *0.249* |
| Biopsy-proven or sub-clinical | 89 (38.2) | 48 (42.1) | 41 (34.5) | 0.85 (0.56 to 1.29) | *0.438* |

Data are number (%). AZA, Azathioprine, MMF, Mycophenolate mofetil.
